# OH formation and H_2_ adsorption at the liquid water–Pt(111) interface†
†Electronic supplementary information (ESI) available: ESI figures and tables as described in the text. This includes a comparison of D_2_O and H_2_O thermo-chemistry, an illustration of how we choose *t*_0_, an overview of all performed MD simulations, corrections used to get reaction Gibbs free energies, movies showing MD trajectories, the structure of the 9*OH and 4O* interfaces, surface coverage and second layer water coverage as a function of *OH, a Pt(111) CV constructed with a shifted *n*_OH_ = 2 reaction energy, DFT energies *versus* work functions, connection between water orientation and work function, autocorrelation functions for DFT energies, work function and water orientation, and Bader charge on a desorbed H atom. See DOI: 10.1039/c8sc02495b


**DOI:** 10.1039/c8sc02495b

**Published:** 2018-07-23

**Authors:** Henrik H. Kristoffersen, Tejs Vegge, Heine Anton Hansen

**Affiliations:** a Department of Energy Conversion and Storage , Technical University of Denmark , 2800 Kgs. Lyngby , Denmark . Email: hhkri@dtu.dk ; Tel: +45 45 25 82 05

## Abstract

The liquid water–Pt(111) interface is studied with constant temperature *ab initio* molecular dynamics to explore the importance of liquid water dynamics of catalytic reactions such as the oxygen reduction reaction in PEM fuel cells. The structure and energetics of hydroxyls formed at the liquid water–Pt(111) interface are found to be significantly different from those of the hydroxyl formed on a bare Pt(111) surface and the hydroxyl formed on a Pt(111) surface with a static water layer. We identify 1/12 ML *OH, 5/12 ML *OH and 2/3 ML *OH as particularly stable hydroxyl coverages in highly dynamic liquid water environments, which – contrary to static water–hydroxyl models – contain adjacent uncovered Pt sites. Atomic surface oxygen is found to be unstable in the presence of liquid water, in contrast to static atomic level simulations. These results give an improved understanding of hydroxide and surface oxide formation from Pt(111) cyclic voltammetry and allow us to draw detailed connections between the electrostatic potential and the interface structure. The study of hydrogen adsorption at the liquid water–Pt(111) interface finds competitive adsorption between the adsorbed hydrogen atoms and water molecules. This does not adhere with experimental observations, and this indicates that the Pt(111) surface has to be negatively charged for a correct description of the liquid water–Pt(111) interface at potentials where hydrogen adsorption occurs.

## Abstract 2

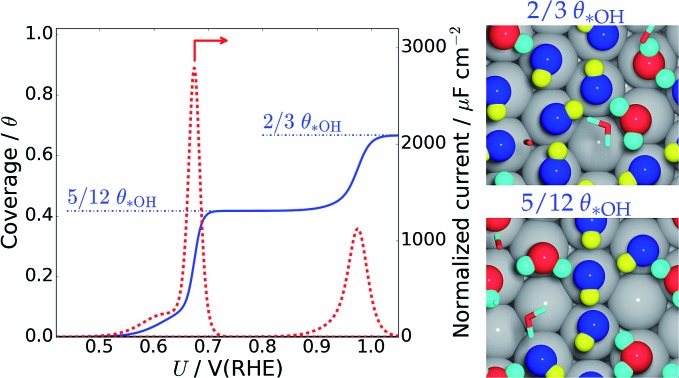
The liquid water–Pt(111) interface is studied with constant temperature *ab initio* molecular dynamics to explore the importance of liquid water dynamics on catalytic reactions such as the oxygen reduction reaction in PEM fuel cells.

## Introduction

Electro-catalysis with aqueous electrolytes has been studied extensively by computational modeling at the atomic level and significant insight has been gained. Modeling has especially been used to identify electro-catalytic reaction mechanisms^1^,^2^ and to propose new electro-catalysts with better catalytic activity.^3^–^8^ However, the vast majority of these studies do not explicitly include the liquid water part of the aqueous electrolyte–electrode interface.^9^ Either water is included as a static layer,^10^–^13^ represented implicitly by a dielectric continuum,^14^,^15^ or water is not included at all.^3^ Still, a more accurate description of the liquid water–electrode interface could be important for a better understanding of important electro-catalytic reactions like hydrogen evolution/oxidation^16^ and oxygen evolution/reduction.^17^ It would also give a better understanding of the accuracy and applicability of both static water–metal interface models and implicit solvation models (dielectric continuum models) previously used. *Ab initio* molecular dynamics (AIMD) captures the dynamics of liquid water and is the method of choice to study liquid water–metal interfaces.^18^–^22^

Platinum electrodes have been studied in great detail,^23^ as they exhibit good electro-catalytic performance for hydrogen evolution/oxidation^24^,^25^ and oxygen reduction,^4^ and are widely used in electro-chemical devices such as PEM fuel cells.^26^ The crystalline Pt(111) surface in contact with a liquid water film is therefore a good starting point for a detailed study, and in this article, we use AIMD to investigate *OH formation (eqn (1)) and H_2_ adsorption (eqn (2)) at the liquid water–Pt(111) interface.

1 *n*_OH_ H_2_O(l) → *n*_OH_ *OH + *n*_OH_ 1/2H_2_(g)

2 *n*_H_ 1/2H_2_(g) → *n*_H_ H*

Our study shows that the water–Pt(111) interfaces are significantly altered by the dynamic description of the water film compared to static water models. At zero and low *OH coverage, the total surface coverage (*OH and H_2_O*) is much lower than that in the static water models. Interfaces with low *OH coverages also have a dynamic structure, where H_2_O molecules regularly get desorbed or adsorbed at the surface on a ∼5 ps time scale at 350 K. When the *OH coverage becomes higher, the dynamic character is diminished, and the total surface coverage becomes larger than in the static water models.

At low H* coverage, the H* species are also affected by the liquid water film, due to competitive adsorption between H* and H_2_O* at the Pt(111) surface. At higher H* coverage, one H atom gets desorbed from the surface, and this affects the liquid water–Pt(111) interface substantially. The desorbed H atom is ionized in the liquid water film and the electron is transferred to the Pt slab. The negative charge in the Pt slab displaces the water molecules from the surface, and hydrogen adsorption is no longer dominated by competitive adsorption with H_2_O*.

## Computational details

**Fig. 1:**
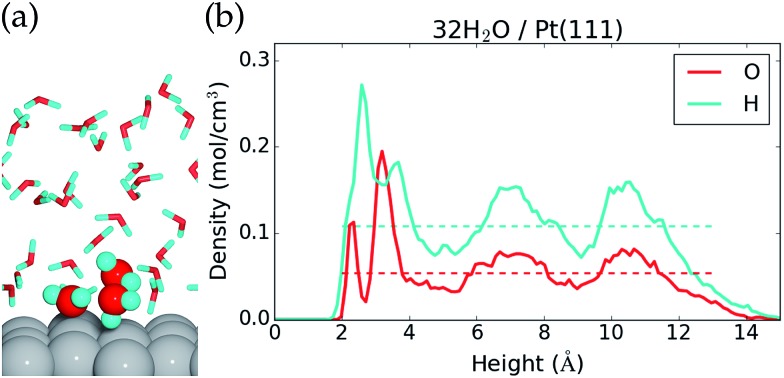
(a) Side view of the last configuration in the MD sampling of the 32H_2_O/Pt(111) reference system and (b) average atomic density as a function of height above the Pt(111) surface. Dashed lines show the O and H atomic densities in bulk liquid water at 350 K.

The liquid water–Pt(111) interface is modeled as (32-*n*_OH_) H_2_O molecules on top of a 3 × 4 orthogonal Pt(111) surface with a thickness of four atomic layers (Fig. 1a). The interface is set up with the ASE program^71^ and studied by constant temperature molecular dynamics (MD) simulations performed in VASP,^27^–^30^ where the temperature is kept around 350 K with a Nose thermostat^31^ and the motion of the atoms is treated classically and propagated with 1 fs time steps. The hydrogen mass is set to 2 g mol^–1^, and all atoms are free to move. Electro-chemistry is usually performed at room temperature with H_2_O (instead of D_2_O), but we assume that our AIMD reaction energies are representative for these conditions as well (justification is presented in the ESI†). We have used higher temperature and deuterium masses in the hope of getting faster thermalisation and better time statistics in the AIMD simulations.

The MD simulations utilize density functional theory (DFT) calculations with a 350 eV energy cutoff plane-wave basis, 2 × 2 × 1 *k*-points, and a spin-paired electron configuration (non-spin polarized). Exchange–correlation effects are approximated by PBE^32^ and the D3 (ref. 33) van der Waals correction. The atomic regions are treated with the PAW formalism, and one, six, and ten valence electrons are included for each H, O, and Pt atom, respectively.

The internal energy (The internal energy (〈*E*〉_*t*_) of a given system is calculated as the time averaged DFT energy (*E*_DFT_) plus the time averaged kinetic energy (*K*) of the MD simulation (eqn (3)). The interface simulations are sampled (*t*–*t*_0_) for more than 30 ps, after being equilibrated (*t*_0_) for at least 1 ps. The equilibration time of each system is determined by monitoring when the internal energy stabilizes (see the ESI†) and *t*_0_ can be more than 20 ps, if the interface structure rearranges substantially.3
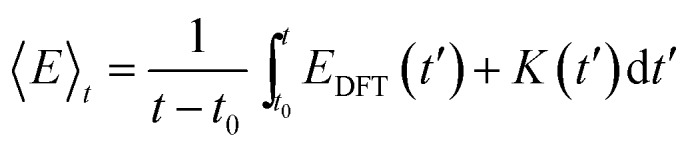



The internal energies are used to calculate the energy cost (Δ*E*) of *OH formation (reaction eqn (1)) and the energy gain (*E*_ad_) of H_2_ adsorption (reaction eqn (2)). Δ*E* is calculated as the internal energy of the interface with *n*_OH_ *OH species and (32-*n*_OH_) H_2_O molecules, plus the internal energy of *n*_OH_/2 H_2_ gas phase molecules, minus the internal energy of the 32H_2_O/Pt(111) reference system (eqn (4)). Adopting the procedure of ref. 34, we add 3/2*k*_B_*T* to the internal energy of gas-phase molecules, because their center-of-mass motions are not included in the MD simulations.4
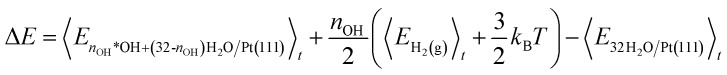



The H_2_ adsorption energy, *E*_ad_, is calculated as the internal energy of the interface with *n*_H_ H* species, minus the internal energy of *n*_H_/2 H_2_(g) and the 32H_2_O/Pt(111) reference system (eqn (5)).5
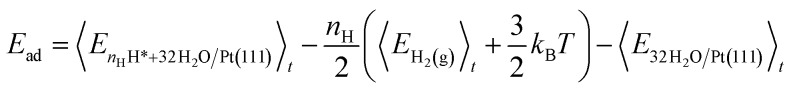



The interfaces are further analyzed using time averaged Bader charges^35^–^37^ and work functions (WFs). Here, the time average is constructed from atomic configurations taken at 1 ps intervals along the MD trajectories, and the DFT calculations have been performed with increased vacuum and a 450 eV energy cutoff. The WF sampling is increased to 0.25 ps intervals for the most stable AIMD simulations with two *OH and five *OH species at the interface to allow for construction of more accurate WF autocorrelation functions.

Obtaining accurate AIMD energies is challenging, and we have identified two main obstacles. Firstly, the thermal fluctuations of interface models are large, and long sampling times^21^ are needed to get average energies that oscillate by less than ±0.05 eV (we estimate ∼30 ps of sampling after equilibration). Secondly, we have observed that AIMD simulations representing the same interface, but initialized from different starting configurations, can differ by up to 0.5 eV in internal energy at the end of the energy sampling. This shows that the water film can become trapped in an unfavorable region of phase space without escaping within 30 ps of simulation. Therefore, simulation of each interface should ideally be run multiple times from different starting points to identify the most stable internal energy. In this paper, we mainly focus on *OH formation at the liquid water–Pt(111) interface, and these simulations have been performed at least twice (the most stable structures are used). Simulations of the interfaces with H* species have been run only once, and these results are therefore more uncertain. Fortunately, most simulations contain several *OH or H* species, which improves the signal to noise ratio and the reported reaction energies per species become more robust. The ESI† contains an overview of all performed simulations. Two recent studies^38^,^39^ have also used AIMD to investigate hydroxyl formation at the liquid water–Pt(111) interface. However, they utilized short equilibration times (≤3 ps) and short sampling times (≤2 ps).

## Results and discussion

### Water–Pt(111) interface

The 32H_2_O/Pt(111) interface without any *OH or H* species is discussed first. Fig. 1 shows a side view of the interface structure (at the end of the MD simulation) and the time averaged O and H atomic densities as a function of height above the Pt(111) surface. The water molecules close to the Pt(111) surface often adopt one of three types of configurations. This is illustrated by three water molecules that have been highlighted in Fig. 1a; one water molecule binds to a surface Pt atom through the O atom, one water molecule is situated further from the surface with one H atom pointing towards the Pt(111) surface, and one water molecule is situated further from the surface with both H atoms forming hydrogen bonds with water molecules in the surface region.

The three highlighted water molecules are examples of one surface bound water molecule and two second layer water molecules, and such species give rise to two peaks in the O atomic density plot at 2.3 Å and 3.2 Å above the Pt surface (Fig. 1b). The area under the two O density peaks corresponds to an average coverage of 0.16 ML from the surface bound water and 0.59 ML from the second layer water (one molecule per surface Pt is equal to 1 ML). The observation of low surface coverage and larger second layer coverage is in agreement with other AIMD simulations of water on Pt(111),^20^,^22^ but different from certain MD simulations based on inter-atomic potentials, which predict close to 1 ML coverage of surface bound water molecules.^40^ Importantly, it also differs from the traditional static water bilayer model, which consists of 1/3 ML surface bound water molecules and 1/3 ML water molecules with H pointing towards the surface.^10^,^41^,^42^ The bilayer model is based on experimental observations of a single water layer on Pt(111) at low temperature in an ultrahigh vacuum^41^ and on DFT calculations conducted with different exchange–correlation functionals.^10^,^42^ This model is therefore well established in the scientific community, even though it may not capture the behavior of liquid water.

The H atomic density in Fig. 1b has a maximum between the first two O atomic density peaks, which comes from hydrogen bonds in the surface region and from H pointing towards the surface. Further from the surface, both the O atomic density and the H atomic density oscillate around the density of bulk water (dashed lines), before the densities reach zero at the liquid water–vacuum interface (∼13 Å above the surface). The density oscillations between 5 Å and 12 Å, where we might have hoped for a better agreement with bulk liquid water, could be an artifact of the relative small computational cell used and/or from the over-structuring of liquid water found at the PBE^43^ and PBE + D3 (ref. 44) level of theory.

### Hydroxyl formation

**Fig. 2:**
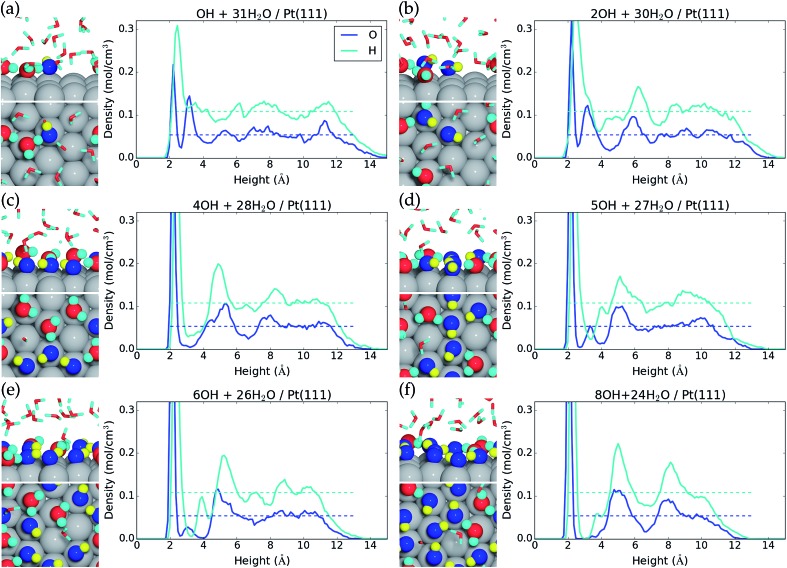
Side view and top view of the interface and average atomic density as a function of height above the Pt(111) surface for (a) one *OH, (b) two *OH, (c) four *OH, (d) five *OH, (e) six *OH, and (f) eight *OH at the liquid water–Pt(111) interface. The atomic configurations are taken at the end of the MD samplings, and surface bound species are depicted with increased radii. *OH is colored blue for O and yellow for H, while H_2_O is colored red for O and cyan for H.

We now investigate formation of hydroxyls at the liquid water–Pt(111) interface by reaction (eqn (1)). The energy cost per *OH species for one, two, four, five, six, eight, and nine *OH species at the surface is reported in Table 1, and the resulting interface structures and atomic densities are shown in Fig. 2. We note that the AIMD energy cost per hydroxyl (Δ*E*/*n*_OH_, Table 1) has to be corrected by –0.17 eV – *TS*_conf_(θ_*OH_) (where *S*_conf_(*θ*) = –*k*_B_(ln[*θ/*(1 – *θ*)] + 1/*θ* ln[1 – *θ*]))^45^ to obtain Gibbs free reaction energies (Δ*G*/*n*_OH_, Table 1) at 298.15 K and 1 atm H_2_(g) (ESI†).^1^,^24^ This correction accounts for the differences in zero point energies and entropies, which are needed, because AIMD treats molecular vibrations classically and do not give entropies directly. We neglect energy corrections from H_2_O co-adsorption, as the H_2_O* coverage changes to a lesser extent than the *OH coverage (Table 1).

**Table 1:** For each *n*_OH_ interface, the *OH coverage (θ_*OH_), surface bound H_2_O* coverage (*θ*_H_2_O*_), energy cost per formed *OH (Δ*E*/*n*_OH_), free energy cost per formed *OH (Δ*G*/*n*_OH_), average Bader charge in the Pt(111) slab (), average Bader charge in the Pt(111) slab (〈*Q*[Pt_48_]]〉_*t*_) and average work function () and average work function (〈WF〉WF) and average work function (〈WF〉_*t*_) are listed. The ) are listed. The 〈WF〉WF) are listed. The 〈WF〉_*t*_ values in parentheses are calculated with 0.25 ps interval sampling instead of 1 ps

*n*_OH_	θ_*OH_	*θ*_H_2_O*_	ΔE/*n*_OH_	ΔG/*n*_OH_	〈*Q*[Pt_48_]]〉_*t*_	〈WF〉WF〈WF〉_*t*_
0	0 ML	0.16 ML			–0.17 *e*	4.3 eV
**1**	**0.08 ML**	**0.19 ML**	**0.87 eV**	**0.61 eV**	**0.26 *e***	**4.7 eV**
2	0.17 ML	0.22 ML	0.89 eV	0.65 eV	0.66 *e*	4.2 eV (4.4 eV)
4	0.33 ML	0.32 ML	0.96 eV	0.74 eV	1.42 *e*	4.6 eV
**5**	**0.42 ML**	**0.33 ML**	**0.87 eV**	**0.66 eV**	**1.79 *e***	**4.1 eV (4.3 eV)**
6	0.50 ML	0.34 ML	0.91 eV	0.71 eV	2.10 *e*	4.6 eV
**8**	**0.67 ML**	**0.25 ML**	**0.97 eV**	**0.78 eV**	**2.63 *e***	**5.5 eV**
9	0.75 ML	0.25 ML	1.02 eV	0.83 eV	2.91 *e*	5.3 eV

Formation of one *OH at the water–Pt(111) interface has an energy cost of 0.87 eV and a free energy cost of 0.61 eV (Table 1). Hereby, the 1/12 ML *OH coverage has the lowest free energy cost per *OH species of the investigated coverages. One *OH at the interface increases the total surface coverage (H_2_O* and *OH) from 0.16 ML to 0.28 ML (Fig. 2a). This increase corresponds to the addition of one *OH species, which is always adsorbed on the surface, and the average adsorption of an additional “0.37” water molecule. Meanwhile, the second water layer is reduced by 1.70 water molecules on average, which corresponds to a reduction from 0.59 ML to 0.45 ML.

On a bare Pt(111) surface, we find that the reaction H_2_O* → *OH + 1/2H_2_(g) has a DFT energy cost of 1.19 eV. Therefore, the presence of H_2_O molecules at the liquid water–Pt(111) interface stabilizes a single *OH species by –0.3 eV more than a single H_2_O* species.

Formation of two *OH species at the water–Pt(111) interface (Fig. 2b) increases the energy cost per *OH to 0.89 eV and the free energy cost to 0.65 eV. The average amount of adsorbed H_2_O* is increased by 0.33H_2_O molecule such that the total surface coverage (H_2_O* and *OH) becomes 0.39 ML. However, the amount of water in the second layer remains the same as with one *OH.

Interestingly, the formation of four *OH at the water–Pt(111) interface (1/3 ML coverage) is found to be unfavorable with an energy cost of 0.96 eV (free energy cost of 0.74 eV) per *OH. The free energy cost is hereby higher for the 1/3 ML *OH coverage than for any other investigated coverages below 2/3 ML. This is quite surprising, since studies (with the RPBE exchange–correlation functional) of *OH formation at a static water bilayer–Pt(111) interface find the 1/3 ML *OH coverage to be the most stable.^11^,^46^ This discrepancy might arise because the static water bilayer is assumed to have a hexagonal ring structure, whereas all our interface structures are very different from the hexagonal ring structure with the exception of the four *OH interface (Fig. 2c). At the four *OH interface, the surface bound H_2_O* and *OH form an undisturbed hexagonal ring pattern. An additional feature of the four *OH interface is that the second water layer is completely depleted (Fig. 2c).

Five *OH at the interface (0.42 ML *OH coverage) is found to have the same low energy cost per *OH (0.87 eV) as the 1/12 ML *OH coverage and a significantly lower free energy cost per *OH (0.66 eV) than both 1/3 ML and 1/2 ML *OH coverages. The resulting interface structure is shown in Fig. 2d. The combined H_2_O* and *OH surface coverage is 0.75 ML, which means that nine out of the 12 surface Pt(111) sites are covered by either H_2_O* or *OH. Interestingly, the remaining three uncovered Pt(111) sites are situated next to each other. This is completely opposite to the hexagonal ring structure found for four *OH species and assumed in the static water bilayer model, where the uncovered Pt(111) sites are always surrounded by six covered Pt(111) sites. The second water layer constitutes a coverage of 0.13 ML at the five *OH interface. The presence of adjacent uncovered Pt(111) sites is important and could facilitate adsorption of species that binds to more than one Pt atom at a time. This could for instance be O_2_* which adsorbs in a bridge configuration between two neighboring Pt sites. The lack of uncovered neighboring Pt sites has been linked to high activation energy for O_2_ dissociation and high over-potential for oxygen reduction.^11^,^46^

Formation of six *OH (1/2 ML coverage) and eight *OH (2/3 ML coverage) at the interface has energy costs of 0.91 eV and 0.97 eV per *OH and free energy costs of 0.71 eV and 0.78 eV per *OH (Table 1). However, if we consider the addition of one or three *OH to the interface that already has five *OH species, the energy costs per *OH become 1.13 eV and 1.13 eV and the free energy costs per *OH become 0.96 eV and 0.97 eV, respectively. The interfaces with six and eight *OH species are therefore effectively equally stable, when compared to the interface with five *OH species. The increase in *OH coverage further modifies the interface structure such that only two adjacent Pt(111) atoms out of 12 are uncovered with six *OH and only a single Pt(111) atom is uncovered with eight *OH species. The second water layer is also further depleted compared to the five *OH interface and is completely gone with eight *OH species at the interface.

The formation of nine *OH species at the interface is very costly with regard to both the free energy cost per *OH (0.83 eV, Table 1) and especially the free energy cost of adding one *OH to the interface that already has eight *OH species (1.24 eV). The structure of the interface with nine *OH is therefore shown in the ESI† and higher *OH coverages have not been considered.

Static water–Pt(111) models studied with the RPBE exchange–correlation functional predict that 1/3 ML O* is more stable than 2/3 ML *OH on the Pt(111) surface.^11^,^47^ We therefore specifically considered conversion of 2/3 ML *OH to 1/3 ML O* through the reaction “8*OH → 4O* + 4H_2_O(l)”. This reaction is found to have an AIMD energy cost of +0.51 eV and an estimated free energy cost of +0.20 eV. The 1/3 ML O* structure is therefore not stable and only shown in the ESI.† In addition, we never observe spontaneous formation of O* species through the reaction 2*OH → O* + H_2_O* at any *OH coverage, which also indicates a non-negligible energy cost for O* formation.^38^ Mixed networks of O*, *OH, and H_2_O* have been reported to increase the stability, and it is possible that O* species exist in such structures.^48^ However, we have not explicitly studied this possibility.

At this point, it is informative to discuss ground state DFT results (with different exchange–correlation functionals) for H_2_O adsorption, *OH formation and O* formation on a bare 3 × 4 orthogonal Pt(111) surface. With our computational setup (PBE and D3 vdW correction), water adsorption (H_2_O(g) → H_2_O*) stabilizes the energy by –0.48 eV, hydroxyl formation (H_2_O* → *OH + 1/2H_2_(g)) costs 1.19 eV and O* formation (*OH → O* + 1/2H_2_(g)) costs 0.48 eV. These reaction energies show that O* formation is less costly than 2*OH formation on the bare Pt(111) surface. The same reactions calculated with the RPBE exchange–correlation functional have the following reaction energies: –0.05 eV, 0.99 eV, and 0.56 eV.^49^ Therefore, our computational setup actually stabilizes O* compared to *OH as seen by our lower energy cost for *OH → O* + 1/2H_2_(g) compared to RPBE. The main difference is in the H_2_O adsorption energy, where we find that H_2_O binds much stronger to the surface, due to both our use of PBE and our inclusion of the D3 van der Waals correction.^50^ The stronger water binding stabilizes the 8*OH interface better compared to the 4O* interface, because the 4O* interface does not allow co-adsorbed water molecules on the surface.

The AIMD simulations also show that the liquid water–Pt(111) interfaces have interesting dynamic differences. The 32H_2_O/Pt(111) interface, without any *OH species, is not particularly dynamic, and only a few events of H_2_O adsorption and desorption happen during the energy sampling. On the other hand, the water–Pt(111) interfaces with one or two *OH species (θ_*OH_ = 0.08 ML and 0.17 ML) are found to be much more dynamic. Here, protons are transferred between adsorbed *H_2_O species and *OH species on a <1 ps timescale indicating a barrier of ∼0.1 eV, and H_2_O molecules get adsorbed and desorbed at the surface on a ∼5 ps timescale indicating that low coverage of *OH species promotes H_2_O adsorption/desorption perhaps by functioning as anchoring points for H_2_O molecules. The dynamics occurring at interfaces with low coverage of *OH means that, over time, an *OH species initially present on the surface can acquire a proton from a surface bound H_2_O* molecule and leave the surface, while H_2_O molecules from the second layer or from the water film can be adsorbed on the surface and subsequently be converted to an *OH species. Finally, when the *OH coverage is increased (θ_*OH_ > 0.17 ML), the surface bound H_2_O* and *OH species form a more detached surface layer and the adsorption/desorption of H_2_O molecules happens less often, whereas proton transfer between adsorbed *H_2_O species and *OH species still occurs frequently. The ESI† contains movies of the MD trajectories to illustrate these phenomena.

### Simulation of Pt(111) cyclic voltammogram

The *OH free energy costs in Table 1 show that certain *OH coverages (for instance 1/12 *OH and 5/12 *OH) are more stable than others. However, the full merit of the free energy costs for *OH formation becomes apparent, when the values are used to approximate the *OH coverage as a function of electrostatic potential. This is done in two steps: first we employ the computational hydrogen electrode model^51^ (eqn (6)) to relate the Gibbs free energy of “H^+^(aq) + e^–^” to the Gibbs free energy of H_2_(g) and the electrostatic potential (*U*) *vs.* the reversible hydrogen electrode (RHE). This is done because H^+^(aq) + e^–^ rather than 1/2H_2_(g) is the actual product species resulting from *OH formation at *U* > 0 V.

6 *G*(H^+^(aq) + e^–^) ≈ 1/2*G*(H_2_(g)) – *eU*

Eqn (6) means that if *OH formation (reaction eqn (1)) has a Gibbs free energy cost of 0.6 eV per *OH (Δ*G*/*n*_OH_, Table 1), the *OH formation will be downhill (Δ*G* < 0) at *U* > 0.6 V. Secondly, we write equilibrium equations between the 32H_2_O/Pt(111) reference system and each *n*_OH_ interface (eqn (7)).7
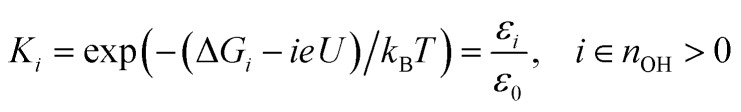
Here, *ε*_*i*_/*ε*_0_ is the ratio of the surface area with *θ*_*i*_ *OH coverage to the surface area with zero *OH coverage and Δ*G*_*i*_ is the total free energy cost of forming *i* *OH species by eqn (1). In addition, probability conservation dictates that 
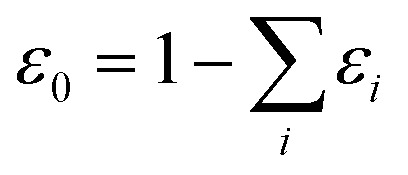
. This treatment is similar to competitive adsorption between different species.^52^

**Fig. 3:**
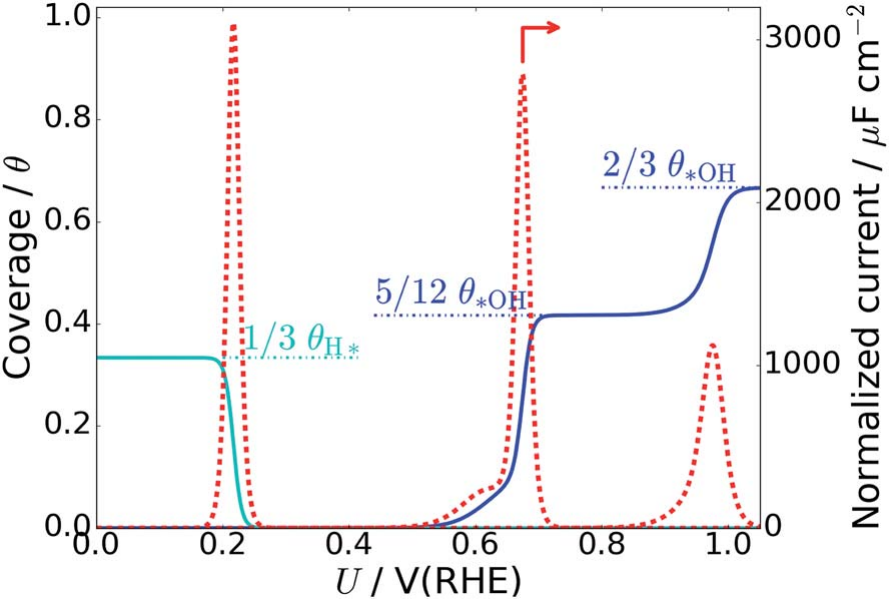
*OH coverage as a function of electrostatic potential (blue curve) and simulated Pt(111) cyclic voltammogram (CV) in the form of the scan rate normalized current (red dashed curve, proportional to d*θ*/d*U*). H* coverage as a function of electrostatic potential (cyan curve) is also included and calculated from Δ*G* for *n*_H_ = 0, 1, 2, 4. The H* coverage jumps directly from 1/3 ML (*n*_H_ = 4) to 0 ML (*n*_H_ = 0) at 0.22 V, and this does not adhere with experimental results, where the H* coverage decreases gradually with increased electrostatic potential.^57^

The average *OH coverage 
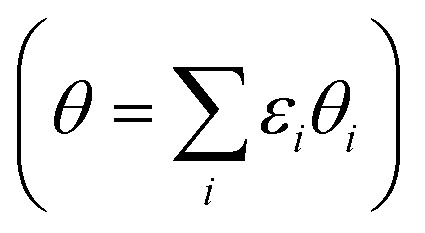
 as a function of electrode potential is shown in Fig. 3. The onset happens around 0.55 V and reaches 1/12 ML *OH at 0.65 V *vs.* RHE, where it jumps directly to 5/12 ML *OH at 0.70 V *vs.* RHE. The 5/12 ML *OH coverage is stable until 0.90 V, where it shifts to 2/3 ML *OH.

As long as the formation of *OH is not kinetically hindered, the change in *OH coverage with potential (d*θ/*d*U*) will be proportional to the scan rate normalized current response in Pt(111) cyclic voltammetry (CV) (dashed red curve in Fig. 3). The normalized current plot has many features in common with the experimentally obtained Pt(111) CV measured in 0.1 M HClO_4_.^53^–^56^ Mainly, a butterfly feature is seen consisting of a 0.6 V *vs.* RHE shoulder peak and a 0.67 V *vs.* RHE sharp peak. In the experimental CV, a similar butterfly feature is situated between 0.6 and 0.8 V *vs.* RHE. The experimental butterfly feature is situated at 0.1 V higher potential and the shoulder peak is bigger, but otherwise the similarities to the simulated CV are very apparent. We note that if the *n*_OH_ = 2 simulation had been –0.04 eV more stable in total energy, it would have contributed to the shoulder peak and the similarities would have been even bigger (this is well within our expected accuracy and a plot with this modification is included in ESI, Fig. S6b†). In addition, the 5/12 ML (0.42 ML) *OH coverage corresponds well to the *OH coverage obtained from integrating the experimental current in the butterfly feature, which is usually stated to be between 0.35 ML^55^,^57^,^58^ and 0.45 ML.^56^,^59^

The next peak in the simulated CV plot is situated at 0.98 V and fits very well with the next current peak after the butterfly feature situated at 1.02 V in the acidic Pt(111) CV,^53^ though, admittedly, the size of our 0.98 V peak is smaller than the experimental 1.02 V peak, which roughly contains the same current as the butterfly feature. The simulated peak is caused by the jump to 2/3 ML *OH, however, since the peak area is too small, it is possible that an undiscovered structure of O* or a mixed O*, *OH, and H_2_O* network with a higher degree of reduction than 2/3 ML *OH could form instead.

The underlying reaction responsible for the experimental 1.02 V current peak is kinetically slow (perhaps irreversible) and has been associated with the formation of O* species at the surface.^53^ This is something we cannot currently corroborate, since 1/3 ML O* is unstable. Instead, our 2/3 ML *OH interface structure suggests another possible explanation for the slow kinetics. At the 2/3 ML *OH coverage, the adsorbed *OH and H_2_O* surface layer is completely detached from the bulk water film; *i.e.* both the O and H atomic density reaches zero at 3 Å (Fig. 2f). This would likely result in slow proton transfer between the surface layer and bulk water and therefore slow kinetics with respect to *OH formation and removal.

It is noteworthy that the simulated CV, related to *OH formation on Pt(111), fits well with the experimental CV measured in an acid electrolyte (0.1 M HClO_4_),^53^–^56^ but does not fit particularly well with Pt(111) CVs measured in a base.^54^,^55^ Our approach accounts for the Nernstian shift due to pH by referring to the reference hydrogen electrode potential, but does not account for the explicit presence of ions. One possibility is therefore that different cations interact differently with *OH species, which has been observed experimentally,^60^,^61^ and further that H^+^(aq) interact so weakly with *OH that the explicit presence of H^+^(aq) is not required in the simulations. However, this interpretation needs additional studies to be confirmed.

### Charge redistribution due to hydroxyl formation

**Fig. 4:**
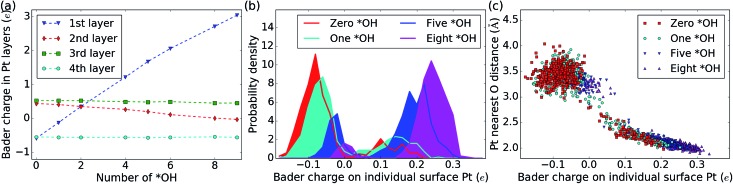
(a) Average Bader charge in each Pt layer as a function of *OH at the interface. The “1st layer” is the Pt layer at the water–Pt(111) interface. (b) Bader charge distribution for the individual surface Pt atoms with different numbers of *OH at the surface. (c) Distance between each individual surface Pt atom and the nearest O atom (in either *OH or H_2_O) plotted *versus* the Bader charge in that Pt atom. Data from every analyzed atomic configuration in the MD trajectories for zero, one, five, and eight *OH are shown in (b) and (c).

Table 1 also contains the average Bader charge in the Pt slab ( also contains the average Bader charge in the Pt slab (〈*Q*[Pt_48_]]〉_*t*_) with increasing *OH coverage. It is seen that the Bader charge in the Pt slab increases very regularly by 0.3 *e* to 0.4 *e* per *OH species at the interface (the corresponding negative charge is present in the adsorbed *OH species). Furthermore, decomposition of the Bader charge in each Pt layer (Fig. 4a) shows that the charge due to the increased amount of *OH species mainly enters the 1st Pt layer (the Pt layer at the water–Pt interface). When the charge in the top layer increases, the charge in the second layer is slightly reduced, while the charges in the third and fourth Pt layers are unaffected. The third and fourth layers are unaffected, because the surface charge is completely screened at a depth of a few angstrom in the Pt metal slab.^62^ As the number of *OH increases beyond *n*_OH_ = 4, the positive charge from each additional *OH species gets smaller and this is visible as a bend of the 1^st^ and 2^nd^ layer curves in Fig. 4a.

The Bader charge in the top Pt layer is not equally distributed on the surface Pt atoms. In Fig. 4b, we plot the distribution of all atomic Bader charge values belonging to surface Pt atoms taken from the MD trajectories with zero *OH (32H_2_O/Pt(111)), one *OH, five *OH and eight *OH species. Interestingly, the atomic Bader charge distribution is divided into two regions: one below 0.05 *e* and one above 0.05 *e*. When the total Bader charge in the surface is increased due to additional *OH species, the Bader charge distributions are shifted upwards and probability density is moved from the lower region to the upper region.

To illustrate the reason for the two regions in the surface Pt Bader charge distribution, we also plot the distance between each surface Pt atom and their nearest O atom (from H_2_O or *OH) as a function of the Bader charge in each Pt atom at every analyzed point in the MD trajectories (Fig. 4c). Pt atoms whose Bader charge is below 0.05 *e* are always far from O atoms; *i.e.* they are not covered by *OH or H_2_O* species, whereas Pt atoms with a Bader charge above 0.05 *e* always have an O atom within ∼2.5 Å, indicating that they are coved by either *OH or H_2_O*. When the surface contains more *OH species, the Bader charges for both covered and uncovered Pt atoms generally become larger, and the Pt–O distances of covered Pt atoms tend to be short.

Certain studies have found a discernible link between the stability of adsorbed *OH species and the electrostatic potential at the interface.^10^,^63^,^64^ These studies apply an external electric field across the Pt slab, which, depending on the direction, stabilizes or destabilizes the dipole associated with the *OH species (H* species are not affected by the external field). In our study, the electrostatic potential at the interface is linked to the WF through the water film.^65^ Therefore, Table 1 contains the time averaged WF ( contains the time averaged WF (〈WF〉WF contains the time averaged WF (〈WF〉_*t*_) for the different interfaces, but unlike the Bader charges, the WFs do not change systematically with the increasing number of *OH species. In fact, the WF is closely linked to the orientation of the water molecules at any given time, and WF(*t*) oscillates by several eV within each MD trajectory.^21^ The The 〈WF〉WF The 〈WF〉_*t*_ value averaged over 1 ps separated data points may therefore not be converged. Indeed, increasing the sampling to 0.25 ps intervals along the MD trajectories for *n*_OH_ = 2 and 5 shifts = 2 and 5 shifts 〈WF〉WF = 2 and 5 shifts 〈WF〉_*t*_ by +0.2 eV and +0.3 eV, respectively (Table 1). Fortunately, these oscillations can also be used to our advantage by plotting the DFT energy *versus* the work function at distinct times (*E*_DFT_(*t*) *versus* WF(*t*)) (ESI, Fig. S7†). This plot should indicate any stabilizing effect of the electrostatic potential on the *OH species, especially with multiple *OH at the interface. We find that the WF can change by 3 eV without any systematic stabilization or destabilization of *E*_DFT_(*t*), even with nine *OH on the surface (Fig. S7c†). Therefore, our MD data do not indicate any *OH stabilization or destabilization due to the electrostatic potential at the interface.

### Hydrogen adsorption

Hydroxyl formation is thermodynamically prohibited at electrostatic potentials below 0.5 V *vs.* RHE, and instead hydrogen may be adsorbed at the liquid water–Pt(111) interface. We therefore discuss dissociative H_2_ adsorption according to reaction eqn (2) in the last part of this paper. We considered one, two, four, six, and eight H* at the 32H_2_O/Pt(111) interface, and the adsorption energy per H* and adsorption free energy per H* are given in Table 2. The adsorption energy per H* species obtained with AIMD has to be corrected by +0.19 eV – *TS*_conf_(*θ*_H*_) to get Gibbs free reaction energies (Δ*G*) at 298.15 K and 1 atm H_2_(g) (ESI†).

**Table 2:** For each *n*_H_ interface, the H* coverage (*θ*_H*_), surface bound H_2_O* coverage (*θ*_H_2_O*_), adsorption energy per H* (*E*_ad_/*n*_H_), adsorption free energy per H* (Δ*G*/*n*_H_), average Bader charge in the Pt(111) slab (), average Bader charge in the Pt(111) slab (〈*Q*[Pt_48_]]〉_*t*_) and average work function () and average work function (〈WF〉WF) and average work function (〈WF〉_*t*_) are listed

*n*_H_	*θ*_H*_	*θ*_H_2_O*_	E_ad_/*n*_H_	ΔG/*n*_H_	〈*Q*[Pt_48_]]〉_*t*_	〈WF〉WF〈WF〉_*t*_
0	0 ML	0.16 ML			–0.17 *e*	4.3 eV
1	0.08 ML	0.15 ML	–0.09 eV	+0.01 eV	–0.12 *e*	4.8 eV
2	0.17 ML	0.14 ML	–0.20 eV	–0.08 eV	–0.12 *e*	4.3 eV
4	0.33 ML	0.13 ML	–0.36 eV	–0.22 eV	–0.12 *e*	4.3 eV
6^*a*^	0.42 ML	0 ML			–0.60 *e*	4.3 eV
8^*a*^	0.58 ML	0 ML			–0.61 *e*	3.7 eV

^*a*^One H is desorbed from the surface during the equilibration and the system has “5H* + H^+^(aq) + e^–^” or “7H* + H^+^(aq) + e^–^” during the energy sampling.

**Fig. 5:**
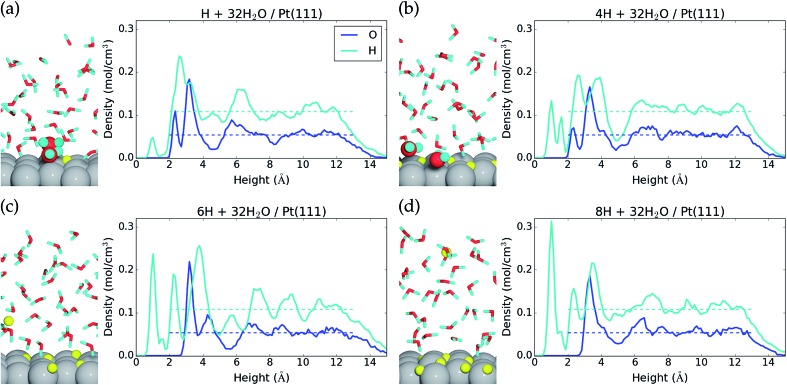
Side view of the interface and average atomic density as a function of height above the Pt(111) surface for (a) one H*, (b) four H*, and (c) 5H* + H^+^(aq) + e^–^, and (d) 7H* + H^+^(aq) + e^–^. The atomic configurations are taken at the end of the MD samplings, surface bound species are depicted with increased radii, and H* and H^+^ are colored yellow.

We find that for one, two, and four H*, the adsorption becomes more favorable with higher coverage (Table 2). This is opposite to the general understanding from experimental Pt(111) cyclic voltammetry, where H* species are not found to have stabilizing interactions; rather they repel each other at higher coverage.^66^–^68^ The mutual repulsion causes the experimental H* coverage to gradually decrease with increasing electrostatic potential,^57^ whereas the mutual attraction in our simulations with *n*_H_ = 0, 1, 2, and 4 causes the H* coverage to jump directly from 1/3 ML (*n*_H_ = 4) to 0 ML (*n*_H_ = 0) at 0.22 V (Fig. 3). On the bare Pt(111) surface, adsorption of one H* in a fcc hollow site^69^ occurs with an adsorption energy of –0.55 eV and adsorption of two H* in neighboring fcc hollow sites occurs with an adsorption energy of –0.54 eV per H*, so there is no inherent attraction between the H* species (with RPBE the adsorption energy per H* is found to be –0.35 eV (ref. 49)). Instead, we believe that the mutual H* attraction in our MD simulations is due to competitive adsorption between H* and H_2_O*.^34^ Adsorption of H* destabilizes and displaces adsorbed H_2_O* resulting in an energy penalty. This effect is weakened with higher H* coverage, where the penalty is divided among more H* species, which effectively leads to attraction between H* species. The competitive adsorption between H* and H_2_O* can also be seen in Fig. 5a and b, which show the interface structures and average atomic densities for one H* (Fig. 5a) and four H* species (Fig. 5b) at the interface. Without any H* species, the coverage of surface bound H_2_O* is 0.16 ML (2.0H_2_O* molecules), but the O atomic density plots in Fig. 5a and b show that the coverage of surface bound H_2_O* is reduced to 0.15 ML (1.8H_2_O* molecules) with one H* and 0.13 ML (1.5H_2_O* molecules) with four H* species. Therefore, the presence of H* species displaces a small amount of surface bound H_2_O*, even though the total coverage of surface bound species is well below 1 ML.

Another interesting observation from Fig. 5a and b is seen in the H atomic density plots. Here, H* species sitting in pure hollow sites with one H* (1.0 Å peak in Fig. 5a) shift to both hollow and top sites with four H* (1.0 Å and 1.6 Å peaks in Fig. 5b).

The origin of the discrepancy between experimental H* adsorption (no attraction) and H* adsorption at our 32H_2_O/Pt(111) interface is perhaps due to an inadequacy in our charge–neutral interface model. Experimentally, there is a shift in the interface H_2_O orientation at 0.35 V,^70^ from H down to O down. The 32H_2_O/Pt(111) interface model matches best with the experimental structure above 0.35 V (O down) (Fig. 1), but H* adsorption occurs below 0.35 V (H down). One way to improve the interface model for H* adsorption could therefore be to lower the electrostatic potential at the interface by negatively charging the Pt(111) surface.

Negative charging of the surface happens spontaneously in the MD simulations that start with either six or eight H* species on the surface. During the initialization period, one H* species is desorbed from the surface to the water film. Here, it is ionized into H^+^(aq) and the e^–^ is transferred to the Pt(111) slab (Bader charge evolution on a desorbed H atom is included in the ESI†). This changes the average Bader charge in the Pt slab from –0.12 *e* (with one, two, and four H*) to –0.61 *e* and –0.61 *e* (Table 2). The water molecules at the interface respond to the negative charge in the surface by desorbing (the O atomic density peak is zero until 3.0 Å in Fig. 5c and d) and reorienting to an H down configuration (a large H atomic density peak is situated at 2.3 Å in Fig. 5c and d).

It is not appropriate to compare the energy of “5H* + H^+^(aq) + e^–^” or “7H* + H^+^(aq) + e^–^” to that of systems without H^+^(aq) + e^–^, but we can obtain the hydrogen adsorption energy of “H_2_(g) + 5H* + H^+^(aq) + e^–^ → 7H* + H^+^(aq) + e^–^”. The adsorption energy per added H* is –0.15 eV, and the adsorption free energy per added H* is +0.05 eV. Given the low values, it can be inferred that at 7/12 ML H* coverages and with negative charge on the Pt surface, repulsive interactions between H* species dominate over competitive adsorption between H* and H_2_O*. This also indicates that the maximum H* coverage is lower than 7/12 ML, as the adsorption free energy becomes positive. This is slightly below the maximum H* coverage of ∼0.7 ML (at *U* → 0 V) actually found in experiments.^49^

## Summary

The study of *OH formation at the 32H_2_O/Pt(111) interface points to several deviations from static water–Pt(111) models; at low (and zero) *OH coverage, the H_2_O* surface coverage is significantly lower than that expected from static models. Of the investigated *OH coverages, we find that the 5/12 ML *OH coverage is very stable and expectedly responsible for the butterfly feature found in Pt(111) cyclic voltammetry with acidic non-adsorbing electrolytes such as HClO_4_. The structure of the 5/12 ML *OH interface has uncovered neighboring Pt sites, and this is likely important for the feasibility of electro-chemical reactions that require two adjacent Pt sites such as O_2_ reduction.

The *OH–H_2_O* structures are highly dynamic with proton hopping occurring on a fast timescale (<1 ps) and H_2_O adsorption or desorption occurring on an ∼5 ps timescale given that the *OH coverage is low (but not zero). Furthermore, we find that the 2/3 ML *OH coverage is more stable than the 1/3 ML O* coverage, and this provides new insight into the structure of the water–Pt(111) interface under very oxidizing conditions (high electric potential).

For H_2_ adsorption, we find that it competes with H_2_O adsorption at low H* coverage leading to effective attraction between H* adsorbates. Above 1/3 ML H*, protons spontaneously desorbs from the surface, which charges the surface negative. This negative surface charge affects the interface structure, where water is desorbed from the surface and orients with H pointing towards the surface. These findings suggest that surface charge is important for a correct description of the water–Pt(111) interface at potentials where H adsorption occurs.

## Conflicts of interest

There are no conflicts to declare.

## Supplementary Material

Supplementary movie

Supplementary movie

Supplementary movie

Supplementary movie

Supplementary information

Supplementary movie

## References

[cit1] Peterson A. A., Abild-Pedersen F., Studt F., Rossmeisl J., Nørskov J. K. (2010). Energy Environ. Sci..

[cit2] Calle-Vallejo F., Koper M. T. M. (2013). Angew. Chem., Int. Ed..

[cit3] Greeley J., Jaramillo T. F., Bonde J., Chorkendorff I., Nørskov J. K. (2006). Nat. Mater..

[cit4] Stamenkovic V., Mun B. S., Mayrhofer K. J. J., Ross P. N., Markovic N. M., Rossmeisl J., Greeley J., Nørskov J. K. (2006). Angew. Chem..

[cit5] Cheng F., Shen J., Peng B., Pan Y., Tao Z., Chen J. (2010). Nat. Chem..

[cit6] Holewinski A., Idrobo J.-C., Linic S. (2014). Nat. Chem..

[cit7] Lim H.-K., Shin H., Goddard W. A., Hwang Y. J., Min B. K., Kim H. (2014). J. Am. Chem. Soc..

[cit8] Back S., Kim H., Jung Y. (2015). ACS Catal..

[cit9] Carrasco J., Hodgson A., Michaelides A. (2012). Nat. Mater..

[cit10] Rossmeisl J., Nørskov J. K., Taylor C. D., Janik M. J., Neurock M. (2006). J. Phys. Chem. B.

[cit11] Tripkovic V., Vegge T. (2017). J. Phys. Chem. C.

[cit12] Hussain J., Jonsson H., Skulason E. (2016). Faraday Discuss..

[cit13] Skulason E., Karlberg G. S., Rossmeisl J., Bligaard T., Greeley J., Jonsson H., Nørskov J. K. (2007). Phys. Chem. Chem. Phys..

[cit14] Sakong S., Groß A. (2016). ACS Catal..

[cit15] Iyemperumal S. K., Deskins N. A. (2017). ChemPhysChem.

[cit16] Zheng Y., Jiao Y., Qiao S., Vasileff A. (2018). Angew. Chem., Int. Ed..

[cit17] Reda M., Hansen H. A., Vegge T. (2018). Catal. Today.

[cit18] Izvekov S., Mazzolo A., VanOpdorp K., Voth G. A. (2001). J. Chem. Phys..

[cit19] Izvekov S., Voth G. A. (2001). J. Chem. Phys..

[cit20] Groß A., Schnur S. (2009). New J. Phys..

[cit21] Sakong S., Forster-Tonigold K., Groß A. (2016). J. Chem. Phys..

[cit22] Ryczko K., Tamblyn I. (2017). Phys. Rev. B.

[cit23] Durst J., Simon C., Siebel A., Rheinländer P. J., Schuler T., Hanzlik M., Herranz J., Hasché F., Gasteiger H. A. (2014). ECS Trans..

[cit24] Nørskov J. K., Bligaard T., Logadottir A., Kitchin J. R., Chen J. G., Pandelov S., Stimming U. (2005). J. Electrochem. Soc..

[cit25] Wendt H., Kolb D. M., Engelmann G. E. and Ziegler J. C., Ullmann’s Encycl. Ind. Chem., 2011.

[cit26] Debe M. K. (2012). Nature.

[cit27] Kresse G., Hafner J. (1994). Phys. Rev. B: Condens. Matter Mater. Phys..

[cit28] Kresse G., Hafner J. (1993). Phys. Rev. B: Condens. Matter Mater. Phys..

[cit29] Kresse G., Furthmüller J. (1996). Comput. Mater. Sci..

[cit30] Kresse G., Furthmüller J. (1996). Phys. Rev. B: Condens. Matter Mater. Phys..

[cit31] Nosé S. (1984). J. Chem. Phys..

[cit32] Perdew J. P., Burke K., Ernzerhof M. (1996). Phys. Rev. Lett..

[cit33] Grimme S., Antony J., Ehrlich S., Krieg H. (2010). J. Chem. Phys..

[cit34] Kristoffersen H. H., Shea J.-E., Metiu H. (2015). J. Phys. Chem. Lett..

[cit35] Bader R. F. W., Atoms In Molecules: A Quantum Theory, Clarendon, Oxford, UK, 1990.

[cit36] Sanville E., Kenny S. D., Smith R., Henkelman G. (2007). J. Comput. Chem..

[cit37] Tang W., Sanville E., Henkelman G. (2009). J. Phys.: Condens. Matter.

[cit38] Ikeshoji T., Otani M. (2017). Phys. Chem. Chem. Phys..

[cit39] Hansen M. H., Nilsson A., Rossmeisl J. (2017). Phys. Chem. Chem. Phys..

[cit40] Limmer D. T., Willard A. P., Madden P., Chandler D. (2013). Proc. Natl. Acad. Sci. U. S. A..

[cit41] Ogasawara H., Brena B., Nordlund D., Nyberg M., Pelmenschikov A., Pettersson L. G. M., Nilsson A. (2002). Phys. Rev. Lett..

[cit42] Michaelides A., Alavi A., King D. A. (2004). Phys. Rev. B: Condens. Matter Mater. Phys..

[cit43] VandeVondele J., Mohamed F., Krack M., Hutter J., Sprik M., Parrinello M. (2004). J. Chem. Phys..

[cit44] Forster-Tonigold K., Groß A. (2014). J. Chem. Phys..

[cit45] Garcia-Araez N., Climent V., Feliu J. (2009). J. Phys. Chem. C.

[cit46] Tripković V., Skúlason E., Siahrostami S., Nørskov J. K., Rossmeisl J. (2010). Electrochim. Acta.

[cit47] Hansen H. A., Rossmeisl J., Nørskov J. K. (2008). Phys. Chem. Chem. Phys..

[cit48] Tian F., Anderson A. B. (2011). J. Phys. Chem. C.

[cit49] Wang S., Petzold V., Tripkovic V., Kleis J., Howalt J. G., Skulason E., Fernandez E. M., Hvolbaek B., Jones G., Toftelund A., Falsig H., Bjorketun M., Studt F., Abild-Pedersen F., Rossmeisl J., Norskov J. K., Bligaard T. (2011). Phys. Chem. Chem. Phys..

[cit50] Carrasco J., Santra B., Klimeš J., Michaelides A. (2011). Phys. Rev. Lett..

[cit51] Nørskov J. K., Rossmeisl J., Logadottir A., Lindqvist L., Kitchin J. R., Bligaard T., Jónsson H. (2004). J. Phys. Chem. B.

[cit52] Chorkendorff I. and Niemantsverdriet J. W., in Concepts of Modern Catalysis and Kinetics, Wiley-VCH Verlag GmbH & Co. KGaA, 2003, pp. 23–78.

[cit53] Gómez-Marín A. M., Clavilier J., Feliu J. M. (2013). J. Electroanal. Chem..

[cit54] van der Niet M. J. T. C., Garcia-Araez N., Hernández J., Feliu J. M., Koper M. T. M. (2013). Catal. Today.

[cit55] Li M. F., Liao L. W., Yuan D. F., Mei D., Chen Y.-X. (2013). Electrochim. Acta.

[cit56] Hitotsuyanagi A., Nakamura M., Hoshi N. (2012). Electrochim. Acta.

[cit57] Stamenkovic V. R., Fowler B., Mun B. S., Wang G., Ross P. N., Lucas C. A., Marković N. M. (2007). Science.

[cit58] Wakisaka M., Suzuki H., Mitsui S., Uchida H., Watanabe M. (2009). Langmuir.

[cit59] Climent V., Gómez R., Orts J. M., Feliu J. M. (2006). J. Phys. Chem. B.

[cit60] Strmcnik D., Kodama K., van der Vliet D., Greeley J., Stamenkovic V. R., Marković N. M. (2009). Nat. Chem..

[cit61] Strmcnik D., Van Der Vliet D. F., Chang K. C., Komanicky V., Kodama K., You H., Stamenkovic V. R., Marković N. M. (2011). J. Phys. Chem. Lett..

[cit62] Newns D. M. (1969). J. Chem. Phys..

[cit63] Karlberg G. S., Rossmeisl J., Nørskov J. K. (2007). Phys. Chem. Chem. Phys..

[cit64] Resasco J., Chen L. D., Clark E., Tsai C., Hahn C., Jaramillo T. F., Chan K., Bell A. T. (2017). J. Am. Chem. Soc..

[cit65] Hansen M. H., Jin C., Thygesen K. S., Rossmeisl J. (2016). J. Phys. Chem. C.

[cit66] Jerkiewicz G. (1998). Prog. Surf. Sci..

[cit67] Koper M. T. M. (2009). Faraday Discuss..

[cit68] Légaré P. (2004). Surf. Sci..

[cit69] Watson G. W., Wells R. P. K., Willock D. J., Hutchings G. J. (2001). J. Phys. Chem. B.

[cit70] Iwasita T., Xia X. (1996). J. Electroanal. Chem..

[cit71] Larsen A., Mortensen J., Blomqvist J., Castelli I., Christensen R., Dulak M., Friis J., Groves M., Hammer B., Hargus C., Hermes E., Jennings P., Jensen P., Kermode J., Kitchin J., Kolsbjerg E., Kubal J., Lysgaard S., Maronsson J., Maxson T., Olsen T., Pastewka L., Peterson A., Rostgaard C., Schiøtz J., Schütt O., Strange M., Thygesen K., Vegge T., Vilhelmsen L., Walter M., Zeng Z., Jacobsen K. J. (2017). J. Phys. Condens. Matter.

