# Supplementary material for: OH formation and H2 adsorption at the liquid water–Pt(111) interface
Source: Chem Sci. 2018 Jul 23;9(34):6912–21. doi: 10.1039/c8sc02495b (PMC6143996; doi:10.1039/c8sc02495b)
Supplement: Supplementary file 5 [file SC-009-C8SC02495B-s005.pdf]

Electronic Supplementary Material (ESI) for Chemical Science.
This journal is © The Royal Society of Chemistry 2018

**Electronic Supplementary Information**

# OH formation and $H_2$ adsorption at the liquid water-Pt(111) interface

Henrik H. Kristoffersen*, Tejs Vegge, and Heine Anton Hansen

Department of Energy Conversion and Storage, Technical University of Denmark, 2800 Kgs. Lyngby, Denmark

*Tel: +45 45 25 82 05; E-mail: hhkri@dtu.dk

## S1. AIMD reaction energies with $D_2O$ at 35O K compared to $H_2O$ at 300 K

MD simulations conducted at 350 K and with $D_2O$ can be used to describe reactions with $H_2O$ at room temperature, if the reactions energies are temperature independent and if the AIMD reaction energies are similar for $D_2O$ and $H_2O$. We therefore show literature reaction enthalpies for $H_2O(l)$ and $D_2O(l)$ dissociation in Table S1. It is seen that the reaction enthalpy for $H_2O(l)$ dissociation into $H_2(g)$ and ½$O_2(g)$ has a very small temperature dependence, i.e. $\Delta H^0$ changes by -2 kJ/mol as the temperature is increased by 62 K (Table S1) from 298.15 K to 360 K. The reaction enthalpies for $H_2O(l)$ dissociation and $D_2O(l)$ dissociation do differ (by 9 kJ/mol, Table S1), but most of the difference (7 kJ/mol) is from changes in zero point energies, which are not included in the AIMD reaction energies, but are corrected for separately.

**Table S1:** Reaction enthalpies (from literature) and changes in zero point energies (ΔZPE, our data) for $H_2O(l)$ and $D_2O(l)$ dissociation.

| Reaction | $\Delta H^0$(298.15 K) | $\Delta H^0$(360 K) | ΔZPE |
|---|---|---|---|
| $H_2O(l) \rightarrow H_2(g) + ½O_2(g)$ | 285.8 kJ/mol [a] | 283.9 kJ/mol [a] | -20.3 kJ/mol |
| $D_2O(l) \rightarrow D_2(g) + ½O_2(g)$ | 294.6 kJ/mol [b] | | -13.5 kJ/mol |

[a] https://janaf.nist.gov/tables/H-063.html

[b] D. D. Wagman, *et al.*, *J. Phys. Chem. Ref. Data*, **1982**, Vol. 11, Suppl. No 2

**S2. Picking $t_0$ to remove internal energy transients at short $t$**

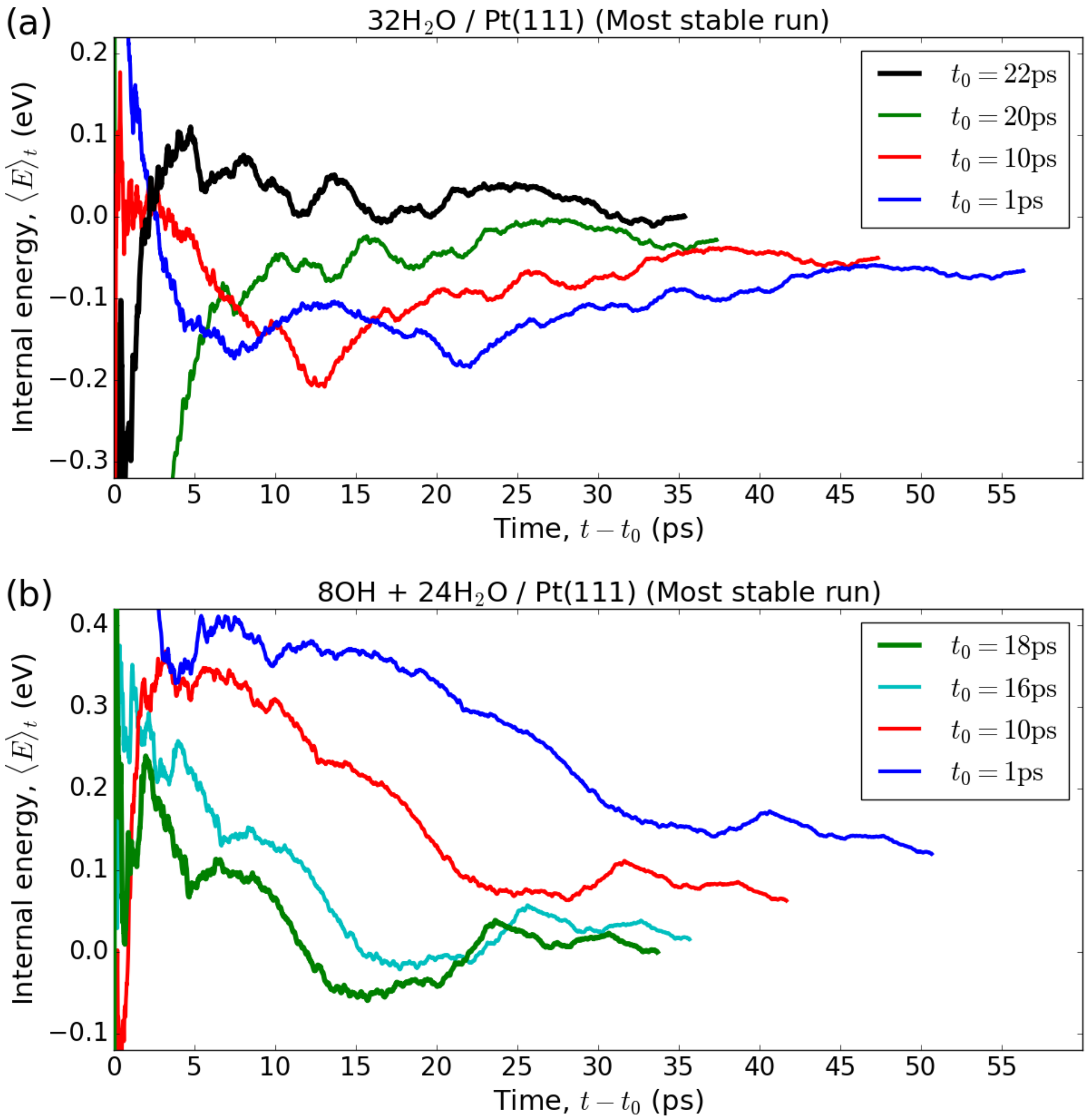


**Figure S1:** Mean internal energy, as a function of elapsed time ($t-t_0$) for the most stable MD simulation of (a) "$32H_2O$ / Pt(111)" and (b) "8OH + $24H_2O$ / Pt(111)". The internal energy transients at short $t$ are removed by increasing $t_0$ to 22 ps for "$32H_2O$ / Pt(111)" and increasing $t_0$ to 18 ps for "8OH + $24H_2O$ / Pt(111)".

## S3. AIMD simulations for hydroxyl formation

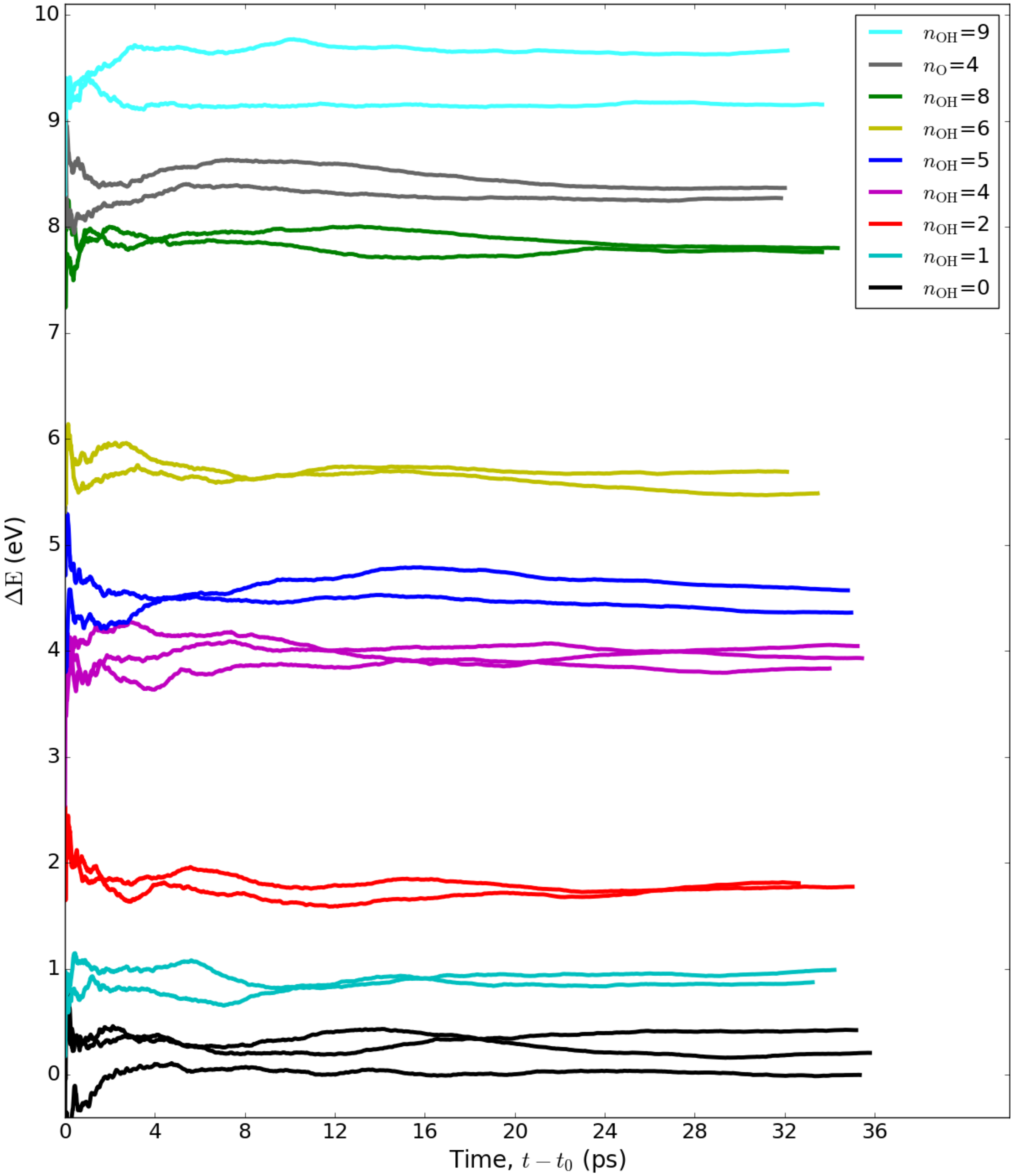


**Figure S2:** Total energy cost of hydroxyl formation (eq 4), as a function of elapsed time ($t-t_0$)

(Running average). The black graphs show the three MD simulations for “$32H_2O$ / Pt(111)” and the most stable simulation is used as the reference (0 eV). Included is also the two MD simulations for $n_{OH}$ = 1, 2, 5, 6, 8, and 9, the three MD simulations for $n_{OH}$ = 4, and the two MD simulation with $n_{O*}$ = 4. From these plots, we estimate that ~30 ps are needed to get time average energies that oscillate by less than ±0.05 eV.

**Table S2:** For each MD simulation of $n_{OH}$ interfaces; energy cost per formed *OH ($\Delta E/n_{OH}$), total energy cost ($\Delta E$), average Bader charge in the Pt(111) slab ($\langle Q[Pt_{48}]\rangle_t$), average work function ($\langle WF\rangle_t$), average number of hydrogen bonds ($\langle HB\rangle_t$) and the chosen thermalisation time ($t_0$) are listed. The $\langle WF\rangle_t$ values in parenthesis are calculated with 0.25 ps interval sampling instead of 1 ps.

| $n_{OH}$ | $\Delta E/n_{OH}$ | $\Delta E$ | $\langle Q[Pt_{48}]\rangle_t$ | $\langle WF\rangle_t$ | $\langle HB\rangle_t$ [a] | $t_0$ |
|---|---|---|---|---|---|---|
| 0 | | 0 eV<br>0.21 eV<br>0.42 eV | -0.17 *e*<br>-0.15 *e*<br>-0.14 *e* | 4.3 eV<br>4.5 eV<br>4.1 eV | 56.0<br>54.7<br>54.5 | 22 ps<br>15 ps<br>13 ps |
| 1 | 0.87 eV<br>0.99 eV | 0.87 eV<br>0.99 eV | 0.26 *e*<br>0.26 *e* | 4.7 eV<br>4.3 eV | 54.9<br>54.6 | 20 ps<br>6 ps |
| 2 | 0.89 eV<br>0.90 eV | 1.78 eV<br>1.81 eV | 0.66 *e*<br>0.65 *e* | 4.2 eV (4.4 eV)<br>4.2 eV | 54.9<br>55.0 | 3 ps<br>16 ps |
| 4 | 0.96 eV<br>0.98 eV<br>1.01 eV | 3.83 eV<br>3.93 eV<br>4.05 eV | 1.42 *e*<br>1.45 *e*<br>1.43 *e* | 4.6 eV<br>4.5 eV<br>4.3 eV | 53.2<br>53.4<br>52.5 | 3 ps<br>10 ps<br>1 ps |
| 5 | 0.87 eV<br>0.91 eV | 4.36 eV<br>4.57 eV | 1.79 *e*<br>1.77 *e* | 4.1 eV (4.3 eV)<br>4.7 eV | 53.9<br>53.5 | 1 ps<br>15 ps |
| 6 | 0.91 eV<br>0.95 eV | 5.49 eV<br>5.69 eV | 2.10 *e*<br>2.09 *e* | 4.6 eV<br>5.0 eV | 52.2<br>52.2 | 11 ps<br>7 ps |
| 8 | 0.97 eV<br>0.97 eV | 7.76 eV<br>7.80 eV | 2.63 *e*<br>2.64 *e* | 5.5 eV<br>5.3 eV | 51.7<br>51.5 | 18 ps<br>11 ps |
| $n_{O*} = 4$ | | 8.27 eV<br>8.37 eV | 2.62 *e*<br>2.61 *e* | 5.1 eV<br>5.2 eV | 51.2<br>51.3 | 67 ps<br>35 ps |
| 9 | 1.02 eV<br>1.07 eV | 9.15 eV<br>9.66 eV | 2.91 *e*<br>2.91 *e* | 5.3 eV<br>5.5 eV | 50.4<br>49.2 | 10 ps<br>6 ps |

[a] The number of hydrogen bonds at a given time is calculated by counting how many of the H atoms are situated between 1.25 Å to 2.40 Å from one of the O atoms.

## S4. AIMD simulations for hydrogen adsorption

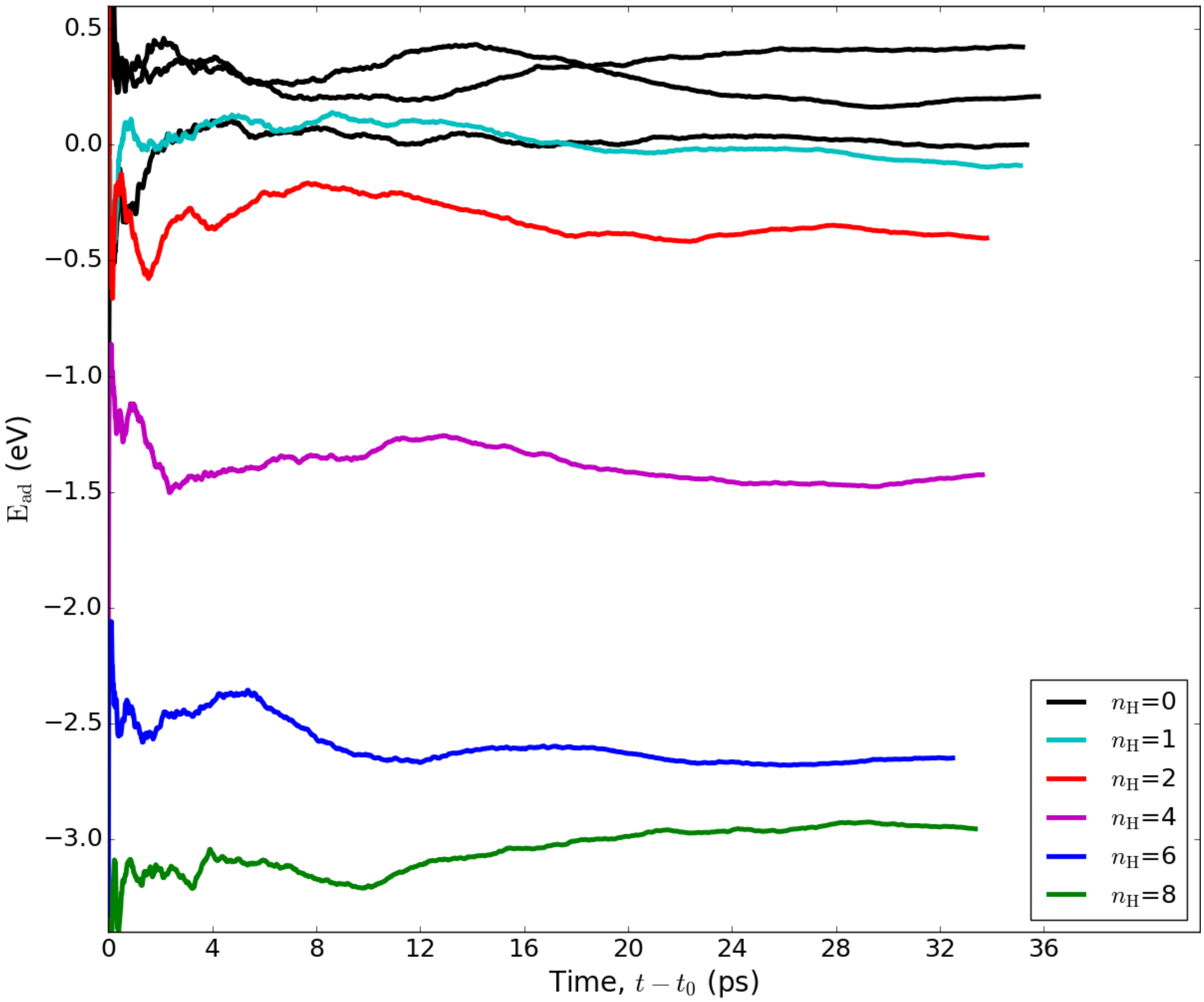


**Figure S3:** Total hydrogen adsorption energies (eq 5), as a function of elapsed time ($t-t_0$). The black graphs show the three MD simulations for "32$H_2O$ / Pt(111)" and the most stable simulation is used as the reference (0 eV). Included is also the single MD simulation for $n_H$ = 1, 2, 4, 6, and 8.

**Table S3:** For each MD simulation of $n_H$ interfaces; adsorption energy per H* ($E_{ad}/n_H$), total adsorption energy ($E_{ad}$), average Bader charge in the Pt(111) slab ($\langle Q[Pt_{48}] \rangle_t$), average work function ($\langle WF \rangle_t$), average number of hydrogen bonds ($\langle HB \rangle_t$) and the chosen thermalisation time ($t_0$) are listed.

| $n_H$ | $E_{ad}/n_H$ | $E_{ad}$ | $\langle Q[Pt_{48}] \rangle_t$ | $\langle WF \rangle_t$ | $\langle HB \rangle_t$ [a] | $t_0$ |
|---|---|---|---|---|---|---|
| 0 | | 0 eV<br>0.21 eV<br>0.42 eV | -0.17 *e*<br>-0.15 *e*<br>-0.14 *e* | 4.3 eV<br>4.5 eV<br>4.1 eV | 56.0<br>54.7<br>54.5 | 22 ps<br>15 ps<br>13 ps |
| 1 | -0.09 eV | -0.09 eV | -0.12 *e* | 4.8 eV | 54.7 | 20 ps |
| 2 | -0.20 eV | -0.40 eV | -0.12 *e* | 4.3 eV | 54.5 | 4 ps |
| 4 | -0.36 eV | -1.43 eV | -0.12 *e* | 4.3 eV | 56.4 | 31 ps |
| 6 [b] | | -2.65 eV | -0.60 *e* | 4.3 eV | 55.1 | 11 ps |
| 8 [b] | | -2.96 eV | -0.61 *e* | 3.7 eV | 54.0 | 38 ps |

[a] The number of hydrogen bonds at a given time is calculated by counting how many of the H atoms are situated between 1.25 Å to 2.40 Å from one of the O atoms.

[b] One H desorbs from the surface during the equilibration and the system has "5H* + $H^+$(aq) + $e^-$" or "7H* + $H^+$(aq) + $e^-$" during the energy sampling.

## S5 Free energy corrections

**Table S4:** Corrections used to get reaction Gibbs free energies, $\Delta G(298.15\text{ K}, 1\text{ atm }H_2) = \Delta E_{AIMD} + \Delta ZPE + p\Delta V - T\Delta S$. The configurational entropy of adsorbed species is approximated by $S_{conf}(\theta) = -k_B(\ln[\theta/(1-\theta)]+1/\theta \ln[1-\theta])$.

| Species | ZPE [a] | -TS [b] | $pV \approx n_{(g)} k_B T$ |
|---|---|---|---|
| $H^*$ | 0.14 eV | -0.007 eV – $TS_{conf}(\theta_{H^*})$ | 0 eV |
| $^*OH$ | 0.28 eV | -0.079 eV – $TS_{conf}(\theta_{^*OH})$ | 0 eV |
| $O^*$ | 0.05 eV | -0.038 eV – $TS_{conf}(\theta_{O^*})$ | 0 eV |
| $H_2O^*$ | 0.49 eV | -0.203 eV – $TS_{conf}(\theta_{H2O^*})$ | 0 eV |
| $O_2$(g) | 0.10 eV | -0.634 eV | 0.026 eV |
| $H_2$(g) | 0.27 eV | -0.404 eV | 0.026 eV |
| $H_2O$(l) | 0.53 eV | -0.216 eV | 0 eV |

[a] Zero point energies (ZPE) are based on frequency peaks obtained from the spectral density of MD trajectory velocity-velocity autocorrelation functions [J. Martí, *J. Chem. Phys.* **1999**, *110* (14), 6876-6886]. The frequencies with deuterium are multiplied by sqrt(2) to get hydrogen frequencies.

[b] Entropy of H*, *OH, and O* is obtained from [A. A. Peterson, *et al.*, *Energy Environ. Sci.* **2010**, *3* (9), 1311–1315], while $H_2O^*$ is calculated in a similar fashion for this project. The entropy of $O_2$(g), $H_2$(g), and $H_2O$(l) is obtained from http://kinetics.nist.gov/janaf/.

### $n\ H_2O(l) \rightarrow n\ \frac{1}{2}H_2(g) + n\ {}^*OH$

$\Delta G(298.15\text{ K}, 1\text{atm }H_2) = \Delta E_{AIMD} + n\,(-0.115\text{ eV} -0.065\text{ eV} + 0.013\text{ eV} - TS_{conf}(\theta_{^*OH}))$

### $n\ H^* \rightarrow n\ \frac{1}{2}H_2(g)$

$\Delta G(298.15\text{ K}, 1\text{atm }H_2) = \Delta E_{AIMD} + n\,( -0.005\text{ eV} -0.195\text{ eV} + 0.013\text{ eV} - Ts_{conf}(\theta_{H^*}))$

### $8^*OH \rightarrow 4O^* + 4H_2O(l)$

$\Delta G(298.15\text{ K}, 1\text{atm }H_2) = \Delta E_{AIMD} + ( +0.08\text{ eV} -0.384\text{ eV}) = \Delta E_{AIMD} -0.304\text{ eV}$

**S6 Movies of the MD trajectories**

Video of the most stable MD trajectory for;

“32$H_2O$ / Pt(111)” (32H2O+Pt111.mpg),

“OH + 31$H_2O$ / Pt(111)” (OH+31H2O+Pt111.mpg),

“2OH + 30$H_2O$ / Pt(111)” (2OH+30H2O+Pt111.mpg),

“5OH + 27$H_2O$ / Pt(111)” (5OH+27H2O+Pt111.mpg), and

“8OH + 24$H_2O$ / Pt(111)” (8OH+24H2O+Pt111.mpg).

The simulation time ($t$-$t_0$) is included in the videos.

## S7 Structure of the nine *OH and four O* interfaces

(a)

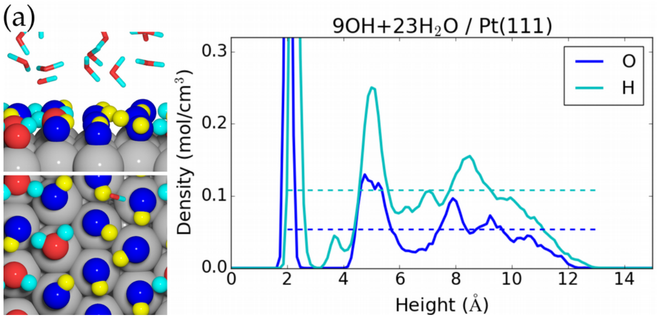


(b)

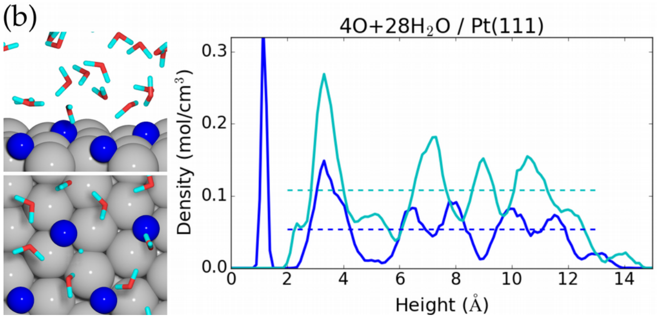


**Figure S4:** Side view and top view of water-Pt(111) interface and average atomic density as a function of height above the Pt(111) surface for (a) nine *OH and (b) four O*. The atomic configurations are taken at the end of the MD samplings and surface bound species are depicted with increased radii. *OH and O* are colored blue for O and yellow for H, while $H_2O$ is colored red for O and cyan for H.

### S8 Coverage of surface bound $H_2O$* and second layer water

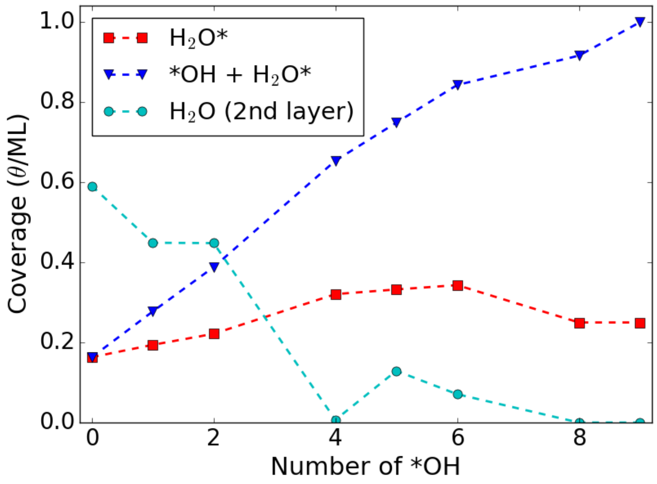


**Figure S5:** Coverage of surface bound water ($H_2O$*), surface bound $H_2O$* plus *OH (*OH + $H_2O$*), and second layer $H_2O$ (2nd layer) for different number of *OH at the liquid water-Pt(111) interface.

## S9 Pt(111) CV without and with shifted ΔE($n_{OH}$=2)

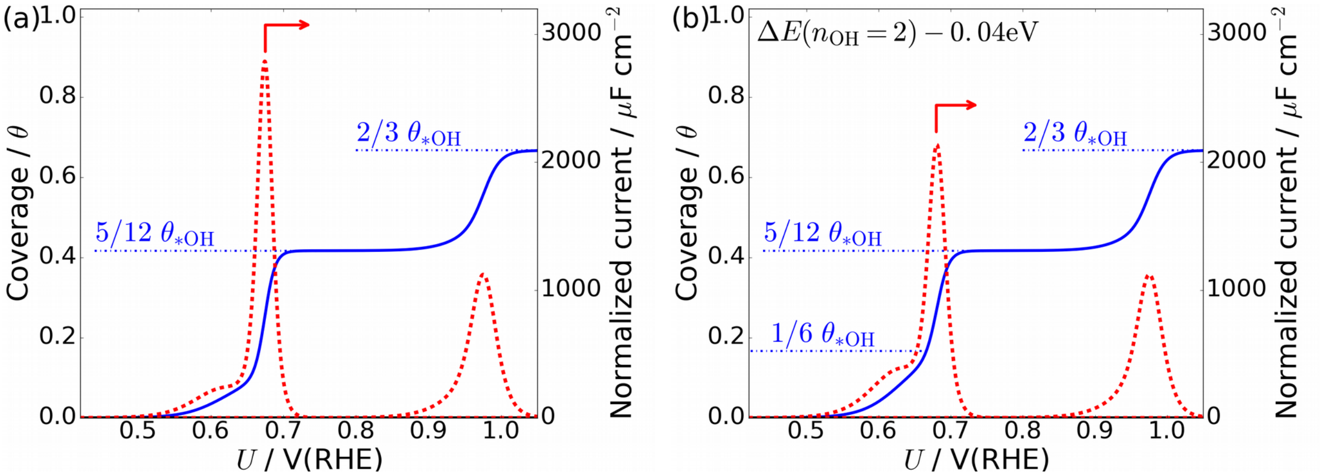


**Figure S6:** *OH coverage as a function of electrostatic potential (blue curve) and simulated Pt(111) cyclic voltammogram (CV) (red dashed curve, proportional to d$\theta$/d$U$) for (a) unmodified energies (Similar to Fig. 3) and (b) with ΔE($n_{OH}$=2) shifted by -0.04 eV. The exact shape of the butterfly feature seen between 0.55 V and 0.70 V in the simulated Pt(111) CV is strongly depended on the total energy costs of forming one, two and five *OH at the interface. Here we show that stabilizing the total energy cost of forming two *OH (ΔE($n_{OH}$=2)) by -0.04 eV allows the 1/6 ML *OH coverage to contribute, before the *OH coverage jumps to 5/12 ML. This makes the shoulder peak more pronounced (it reaches a scan rate normalized current of 500 $\mu$F cm$^{-2}$) and the sharp peak less pronounced (it reaches 2000 $\mu$F cm$^{-2}$). With this -0.04 eV shift in ΔE($n_{OH}$=2), the agreement between the simulated CV and experimental CV becomes better [Gómez-Marín, A. M.; Clavilier, J.; Feliu, J. M., *J. Electroanal. Chem.* **2013**, *688*, 360–370].

## S10 DFT energy vs work function

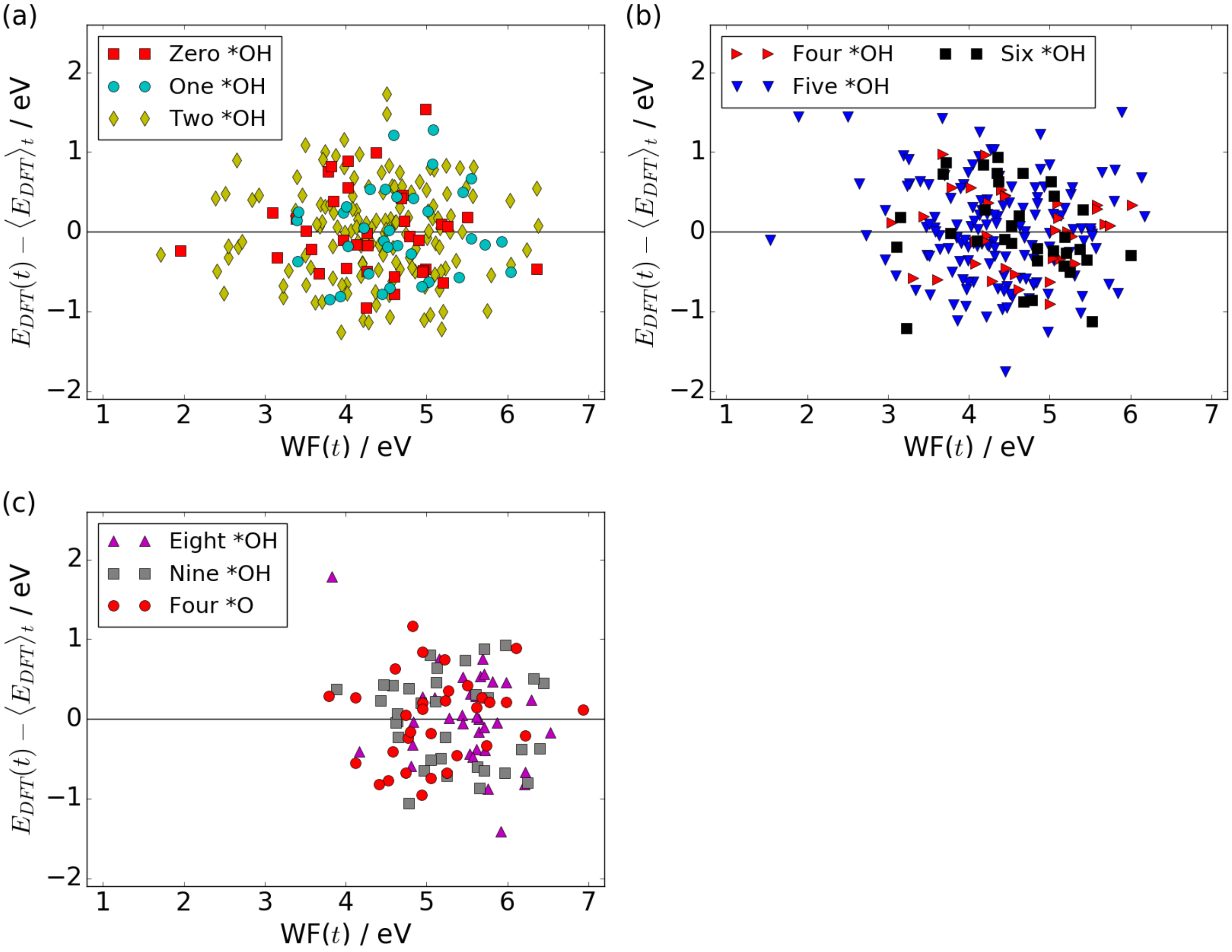


**Figure S7:** Plots of $E_{\mathrm{DFT}}(t)$ versus WF($t$) for $n_{\mathrm{OH}}$ = 1, 2, 5, 6, 8, 9 and $n_{\mathrm{O*}}$ = 4. The data points represent atomic configurations taken at 1 ps or 0.25 ps intervals along the MD trajectories. The WF($t$) can change by 3 eV without any systematic stabilization or destabilization of $E_{\mathrm{DFT}}(t)$, even with nine *OH on the surface. Therefore, our MD data does not indicate any *OH stabilization or destabilization due to the electrostatic potential at the interface.

## S11 Water orientation vs work function

In an attempt to approximate the water orientation by a single geometric quantity, we define $\delta\rho_z(t)$ as the sum over the z-coordinates of H atoms divided by 2, minus the sum over the z-coordinates of O atoms.

$$\delta\rho_z(t) \equiv \frac{1}{2}\sum_{j\in \mathrm{H}_{atoms}} z_j(t) - \sum_{i\in \mathrm{O}_{atoms}} z_i(t)$$

Hereby, a shift in $\delta\rho_z(t)$ over time will indicate a change in the average water orientation. The quantify $\delta\rho_z(t)$ is of course also proportional to the dipole in the z direction originating from the water layer assuming that H atoms have half positive charge and O atoms have one negative charge. Therefore, $\delta\rho_z(t)$ could be related to WF(t). Figure S8 shows the relationship between WF($t$) and $\delta\rho_z(t)$ for the most stable "2OH+ 30$H_2O$ / Pt(111)" and "5OH+ 27$H_2O$ / Pt(111)" AIMD simulations and it is clear that there is a strong correlation. When $\delta\rho_z(t)$ is larger than the average value, i.e. when more water molecules have hydrogen pointing away from the surface, WF($t$) is very low. Opposite, when $\delta\rho_z(t)$ is low because more water molecules have hydrogen pointing toward the surface, WF($t$) is very high.

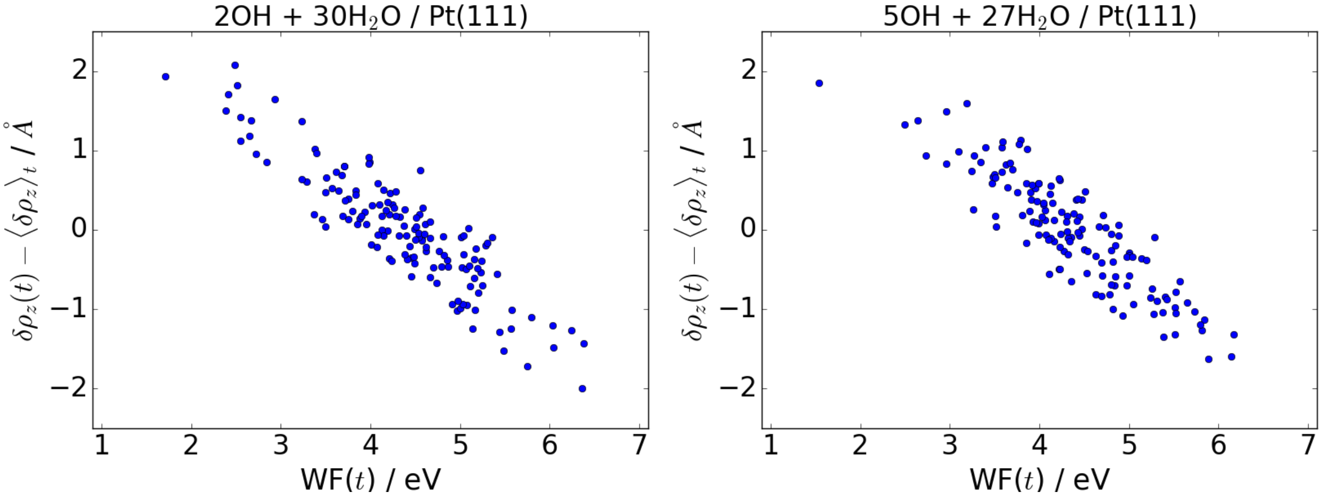


**Figure S8:** Plots of $\delta\rho_z(t)$ versus WF($t$) for $n_{\mathrm{OH}}$ = 2 and 5. The data points represent atomic configurations taken at 0.25 ps intervals along the MD trajectories.

## S12 Autocorrelation functions

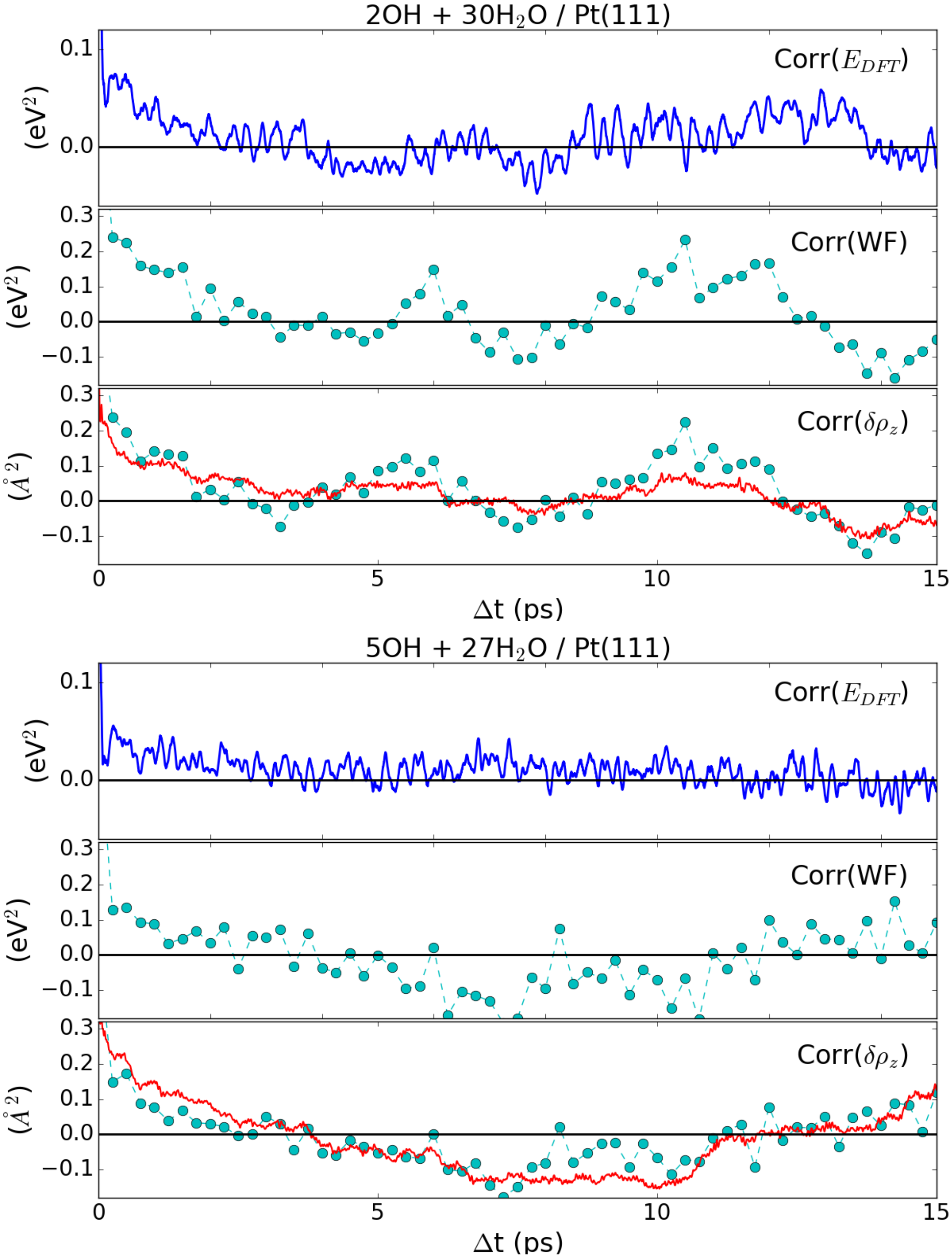


**Figure S9:** Time correlation functions for DFT energy, work function, and $\delta\rho_z(t)$ calculated for the most stable "2OH+ 30H$_2$O / Pt(111)" and "5OH+ 27H$_2$O / Pt(111)" AIMD simulations. The autocorrelation functions are calculated as$\mathrm{Corr}(A)=\langle A(t)A(t+\Delta t)\rangle-\langle A(t)\rangle\langle A(t+\Delta t)\rangle$. Solid lines make

use of the full MD trajectory data, while circles+dashed lines are calculated from data points taken at 0.25 ps intervals along the MD trajectories.

The main observation is that it may require a time-shift (Δt) of ~2 ps to remove the short time correlation in both DFT energy, work function, and $\delta\rho_z(t)$. In addition, both interfaces are subject to very long time-shift correlations. The time-shifts are not the same for the two interfaces, where "2OH+ 30$H_2O$ / Pt(111)" is oscillating with a period of Δt ≈ 5 ps and "5OH+ 27$H_2O$ / Pt(111)" is oscillating with a period of Δt ≈ 15 ps. We speculate that deference could be related to how fast water molecules on the surface or in the liquid water film can respond to the very fast proton transfer dynamics occurring on the surface.

**S13 Bader charge of a desorbing H atom**

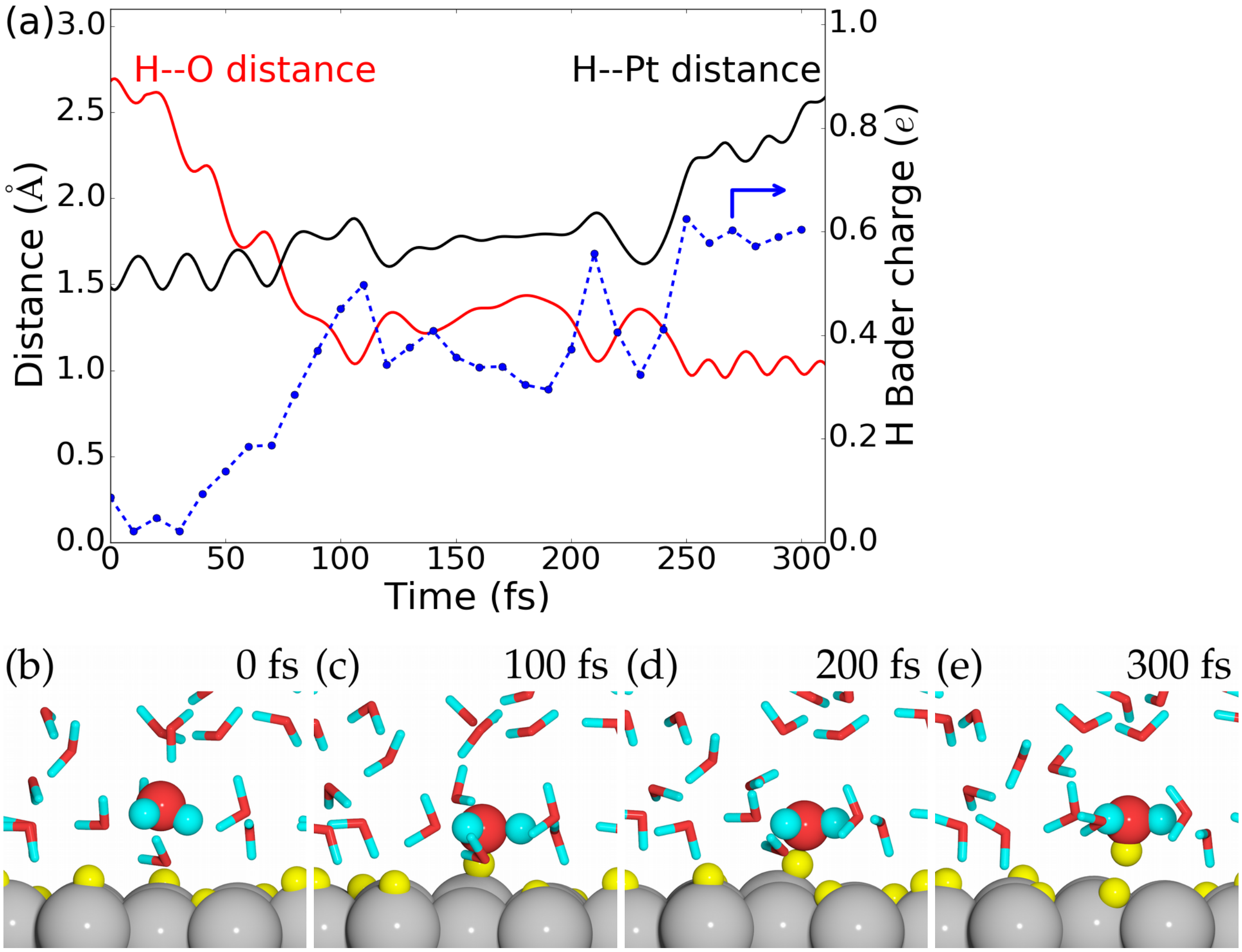


**Figure S10:** (a) Bader charge and distances along 300 fs, where the H atom desorbs into the water film, as observed in the 6H + $32H_2O$ / Pt(111) simulation. Between 0 fs and 100 fs, the water molecule approaches the adsorbed H atom (H--O distance decreases, red curve). In the same period, the Bader charge (dashed blue) on the desorbing H atom increases from ~0 *e* to 0.4 *e*. Between 100 fs and 250 fs, the H atom is both connected to the Pt atom in the surface and the O atom in water. At 250 fs, the H atom detaches from the Pt atom (H--Pt distance increases, black curve) and the Bader charge on the H atom is increased to ~0.6 *e*. Atomic configuration at (b) 0 fs, (c) 100 fs, (d) 200 fs, and (e) 300 fs.
